# Traditional Chinese medicine for type 2 diabetes with metabolic-associated steatotic liver disease: unified effects and design thresholds

**DOI:** 10.3389/fendo.2026.1740126

**Published:** 2026-03-03

**Authors:** Youfang Liu, Bibi Wang, Liting Wang, Haozhe Xiong, Hua Zhang, Yang Cheng

**Affiliations:** 1 Institute of Liver Diseases, Shuguang Hospital, Shanghai University of Traditional Chinese Medicine, Shanghai, China; 2 China–Japan Friendship Hospital, Beijing University of Chinese Medicine, Beijing, China

**Keywords:** interpretable machine learning, knowledge graph, meta-analysis, metabolic-associated steatotic liver disease, traditional Chinese medicine, type 2 diabetes mellitus

## Abstract

**Objective:**

This study aims to reorganize randomized controlled trials (RCTs) of traditional Chinese medicine (TCM) for adults with type 2 diabetes mellitus (T2DM) and metabolic-associated steatotic liver disease (MASLD; including legacy NAFLD) into a clinical evidence-anchored knowledge graph (KG) and harmonize effect semantics (“unified effects”) to support endpoint- and design-aware evidence navigation.

**Methods:**

We systematically reviewed RCTs (2015–2025). Effect direction and scale were unified using a prespecified rule (treatment effect [TE] >0 indicates improvement). The prespecified primary endpoints maximizing cross-trial comparability were alanine aminotransferase (ALT), triglycerides (TG), Homeostatic Model Assessment of Insulin resistance (HOMA-IR), and controlled attenuation parameter (CAP); aspartate aminotransferase (AST) was retained for robustness. Metabolic endpoints were synthesized at the 12-week timepoint, while imaging endpoints (CAP and liver stiffness measurement [LSM]) were synthesized within a prespecified 8- to 24-week window. Trials were stratified as add-on versus mixed, with primary efficacy inferences based on add-on trials with balanced background Western medicine. Evidence was synthesized using REML-based random-effects meta-analysis (reporting prediction intervals) and weighted meta-regression. Risk of bias was assessed using RoB 2 and certainty of evidence using GRADE.

**Results:**

A total of 95 trials were included (*n* = 8,813; follow-up 2–48 weeks; predominantly add-on). The KG linked intervention categories (classic formulas, custom formulas, and Chinese patent medicines) to recurrent syndrome/symptom patterns; *Salvia miltiorrhiza* emerged as a central herb-layer hub. In add-on trials, pooled effects for ALT, AST, HOMA-IR, and TG were directionally favorable, but heterogeneity was substantial and prediction intervals for biochemical endpoints were often wide and crossed the null. CAP showed a comparatively more reproducible short-term imaging signal than LSM. Meta-regression suggested hypothesis-generating design patterns in which estimate stability tended to improve with larger per-arm sample sizes (≈≥40–50) and longer follow-up (≈≥12–16 weeks). RoB 2 ratings were predominantly “some concerns,” and GRADE certainty was commonly downgraded for inconsistency and/or imprecision.

**Conclusion:**

In adults with T2DM and MASLD, add-on trials show directionally favorable pooled biochemical/metabolic changes after unified effect harmonization, but uncertainty remains substantial. CAP may be a more reproducible short-term imaging endpoint than LSM. Evidence-derived design patterns should be interpreted as hypothesis-generating rather than causal thresholds.

**Systematic Review Registration:**

https://www.crd.york.ac.uk/prospero/, identifier CRD420251167450.

## Introduction

Metabolic dysfunction-associated steatotic liver disease (MASLD), formerly non-alcoholic fatty liver disease (NAFLD), is highly prevalent. Its progression relates to insulin resistance, lipotoxicity, chronic low-grade inflammation, and gut–liver axis dysregulation (1, 2). Type 2 diabetes mellitus (T2DM) frequently coexists with MASLD, mutually aggravating the disease course and risks of fibrosis, cirrhosis, and cardiometabolic events (3, 4). Epidemiological evidence suggests that MASLD affects approximately two-thirds of adults with T2DM, underscoring the substantial scale of this comorbidity in clinical practice. Beyond prevalence, this coexistence is clinically consequential: metabolic dysregulation and insulin resistance promote hepatic steatosis and inflammation, while MASLD is associated with increased risks of fibrosis progression, cirrhosis, and adverse cardiometabolic events among patients with diabetes (5–7). Although quantitative imaging—controlled attenuation parameter (CAP) and liver stiffness measurement (LSM)—supports noninvasive assessment, evidence-based therapeutic strategies for T2DM with MASLD remain constrained by limited indications, population heterogeneity, and inconsistent endpoints (4, 8, 9).

Traditional Chinese medicine (TCM) offers multimodal, phenotype-oriented care potentially relevant to T2DM with MASLD, with therapeutic targets spanning insulin resistance, dyslipidemia, low-grade inflammation, and the gut–liver–bile acid axis. In practice, classical formulas, custom formulas, Chinese patent medicines, and single herbs are commonly framed by pattern elements such as phlegm-dampness, damp-heat, liver depression, and spleen deficiency (10, 11). However, translation of existing RCT evidence into actionable guidance is hindered by methodological barriers: trials vary widely in background therapy (add-on vs. mixed designs), intervention complexity, follow-up duration, and endpoint selection; endpoint direction/scale handling is often inconsistent; and analyses frequently stop at pooled averages without systematically examining effect modifiers, practical design thresholds, or integrated efficacy–safety reporting within a prescription–syndrome/symptom–outcome–AE evidence structure (12, 13).

We aimed to reorganize randomized controlled trials’ (RCTs) evidence into a prescription–syndrome/symptom–outcome–AE knowledge graph, synthesize effects using random-effects meta-analysis, quantify design thresholds via weighted meta-regression, and derive phenotype-by-target prescribing implications using clustering and interpretable machine learning. To improve interpretability and reproducibility, we implemented an RCT-anchored, multi-layer framework integrating (i) knowledge graph-based evidence organization, (ii) endpoint standardization, (iii) design-stratified random-effects synthesis, and (iv) threshold/moderator assessment via weighted meta-regression and interpretable machine learning.

Specifically, we introduce two methodological concepts to standardize and interpret the evidence. First, “unified effects” denotes the harmonization of effect direction and scale across heterogeneous endpoints by prespecifying that treatment effect (TE) >0 indicates improvement, thereby enabling cross-endpoint comparability and yielding a coherent “outcome fingerprint.” Second, “design thresholds” refer to empirically derived ranges of sample size and follow-up duration (identified via weighted meta-regression and corroborated by the machine learning component) that are associated with more stable and more reproducible effect estimates.

## Methods

### Reporting and registration

This review followed PRISMA 2020 guidelines; the protocol was registered in PROSPERO (CRD420251167450).

### Literature search

**Figure 1:**
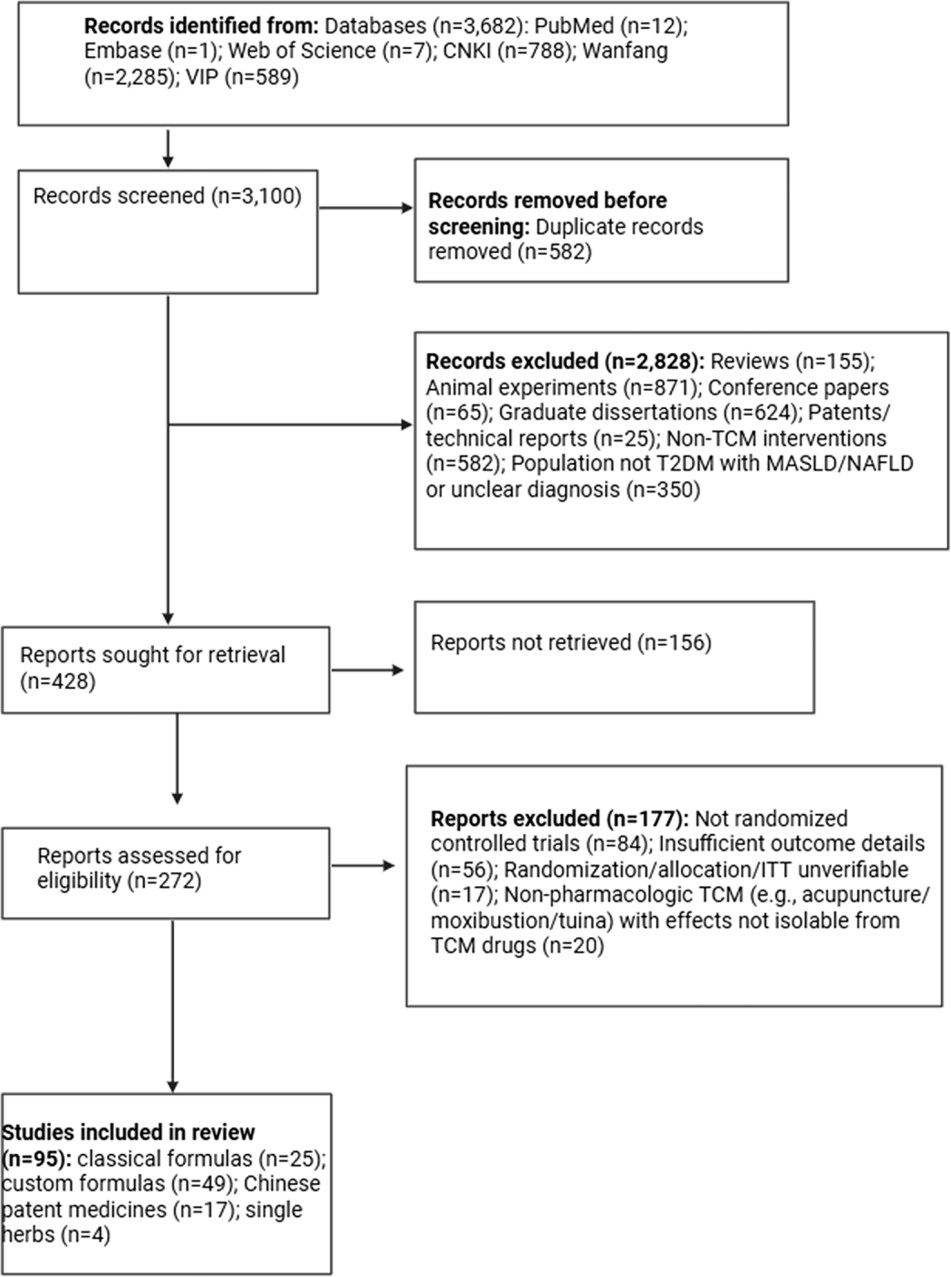
PRISMA 2020 flow diagram for study selection.

We systematically searched PubMed, Embase, Web of Science, China National Knowledge Infrastructure (CNKI), Wanfang Data, and VIP Database for studies published from January 2015 to August 2025. A search strategy combining subject headings and free-text terms was adopted, with core concepts including “NAFLD/MASLD”, “T2DM”, and “traditional Chinese medicine/Chinese herbal medicine/herbal formula”. The reference lists of the included studies were screened manually, and citation tracking was performed (**Figure 1**). Full search strategies and the PRISMA 2020 checklist are provided in **Supplementary Material 1**.

### Eligibility criteria

The inclusion criteria were defined as follows: for study design, RCTs; regarding the study population, adults diagnosed with T2DM complicated by MASLD (including legacy NAFLD terminology; NASH treated as a progressive subtype when explicitly reported); for interventions and comparators, the intervention group received TCM interventions including classical formulas, custom formulas, Chinese patent medicines, or single herbs (with or without concomitant Western medicine), while the control group received conventional Western medicine, placebo, standard care, or lifestyle intervention; for outcomes, at least one prespecified primary outcome was extractable, including alanine aminotransferase (ALT), aspartate aminotransferase (AST), Homeostatic Model Assessment for Insulin Resistance (HOMA-IR), triglycerides (TG), controlled attenuation parameter (CAP), liver stiffness measurement (LSM), and high-density lipoprotein cholesterol (HDL-C). The exclusion criteria included non-RCT study designs, non-pharmacological TCM therapies (e.g., acupuncture, moxibustion), unclear diagnostic criteria for T2DM or MASLD/NAFLD, missing or non-convertible primary outcome data, duplicate publications or overlapping datasets, and non-journal publications (e.g., conference abstracts, dissertations). To ensure standardized reporting, eligibility criteria were explicitly mapped to the PICOS framework: participants (P), adults with T2DM and MASLD/NAFLD; interventions (I), TCM therapies (classical/custom formulas, Chinese patent medicines, or single herbs/extracts) with or without concomitant Western medicine; comparators (C), placebo, standard care, lifestyle intervention, or Western medicine; outcomes (O), at least one prespecified endpoint extractable (primary: ALT, TG, HOMA-IR, CAP; robustness/secondary: AST, HDL-C, LSM); and study design (S), parallel-group randomized controlled trials.

### Data extraction and standardization

Two reviewers independently extracted and cross-checked the data, with discrepancies resolved by arbitration from a third reviewer. At the intervention layer, intervention types and standardized names/codes of formulas were unified; herbal ingredients were aligned with standard names and Latin binomials specified in the Chinese Pharmacopoeia, with syndrome-based modifications annotated (14). The terminologies for TCM syndromes/symptoms were standardized, and unreported items were recorded as “NR” (15, 16). Study designs were categorized into two types: add-on indicates that both arms received background Western medicine (WM) of comparable drug classes and doses, with TCM added only to the intervention arm; and mixed indicates unequal WM intensity between arms (e.g., WM only in one arm or drug class/dose disparity). When reporting was ambiguous, we defaulted to “mixed” and ran design-stratified sensitivity analyses.

To strengthen the control of confounding by concomitant Western medicine (WM), we prespecified a trial-design stratification and documented background-therapy information at the trial level (**Supplementary Material 2**, **Supplementary Table 1**). Trials were categorized as add-on versus mixed (**Supplementary Table 1**, column BA: Design_stratum). Add-on indicates that both arms received comparable background WM (same drug class and dosing rules as reported), with TCM added only to the intervention arm. Mixed indicates that background WM could differ between arms (e.g., WM used in only one arm or drug class/dose disparity) or was not explicitly standardized. For mixed trials, we further coded WM background comparability as balanced/unclear/imbalanced based on extracted WM-related fields (e.g., whether WM was used, WM names, comparator regimen/type/dose) and report this coding in **Supplementary Table 1** (column AZ: WM_comparability). When reporting was insufficient to judge WM balance, comparability was coded as unclear; such trials were retained in the mixed stratum and addressed via prespecified design-stratified exploratory/sensitivity analyses.

To ensure transparent diagnostic reporting and consistent terminology, we extracted trial-level criteria/definitions for T2DM and MASLD/NAFLD (including legacy NAFLD/NASH wording) and coded ascertainment methods. Specifically, T2DM criteria and MASLD/NAFLD criteria were captured in **Supplementary Material 2**, **Supplementary Table 1** (columns N and O), and imaging modality and overall ascertainment summaries were captured in **Supplementary Table 1** (columns BP and BQ). These fields were used for descriptive reporting (**Table 1**) and to support trial-level auditability of diagnostic definitions across studies.

**Table 1:** Summary characteristics of included RCTs in adults with T2DM and MASLD (*n* = 95).

Characteristic	Overall (*n* = 95)	Add-on design (*n* = 82)	Mixed design (*n* = 13)
Total participants, *N*	8,813	7,266	1,547
Follow-up duration (weeks)	2–48; 12 [12–12]	2–48; 12 [12–12]	4–24; 12 [12–16]
Sample size per arm	20–180; 40 [33–54]	20–100; 40 [32–52]	30–180; 40 [38–60]
Country/region	China: 93 (97.9%); other: 2 (2.1%)	China: 80 (97.6%); other: 2 (2.4%)	China:13 (100.0%); other: 0 (0.0%)
Ascertainment (any)	Guideline: 95 (100.0%)	Guideline: 82 (100.0%)	Guideline: 13 (100.0%)
Imaging modality reported	US: 75 (78.9%); CAP: 10 (10.5%); liver CT: 6 (6.3%); LSM: 2 (2.1%); MRI-PDFF: 1 (1.1%); ¹H-MRS: 1 (1.1%)	US: 64 (78.0%); CAP: 9 (11.0%); liver CT: 5 (6.1%); LSM: 2 (2.4%); MRI-PDFF: 1 (1.2%); ¹H-MRS: 1 (1.2%)	US: 11 (84.6%); CAP: 1 (7.7%); liver CT: 1 (7.7%); LSM: 0 (0.0%); MRI-PDFF: 0 (0.0%); ¹H-MRS: 0 (0.0%)
Background therapy reported	Yes: 91 (95.8%); no/unclear: 4 (4.2%)	Yes: 79 (96.3%); no/unclear: 3 (3.7%)	Yes: 12 (92.3%); no/unclear: 1 (7.7%)
Comparator type	WM/standard care: 87 (91.6%); placebo: 3 (3.2%); other: 5 (5.3%)	WM/standard care: 78 (95.1%); placebo: 3 (3.7%); other: 1 (1.2%)	WM/standard care: 9 (69.2%); placebo: 0; other: 4 (30.8%)
TCM intervention category	Classical: 25 (26.3%); custom: 49 (51.6%); patent: 17 (17.9%); single: 4 (4.2%)	Classical: 23 (28.0%); Custom: 45 (54.9%); patent: 10 (12.2%); single: 4 (4.9%)	Classical: 2 (15.4%); custom: 4 (30.8%); patent: 7 (53.8%); single: 0
TCM syndrome/pattern reported	Yes: 60 (63.2%); no: 3 (3.2%); unclear/NR: 32 (33.7%)	Yes: 52 (63.4%); no: 3 (3.7%); unclear/NR: 27 (32.9%)	Yes:8 (61.5%); no: 0; unclear/NR: 5 (38.5%)
Baseline age (years)	Reported 89/95; 37.70–69.88; 54.18 [49.72–57.66]	Reported 77/82; 37.70–69.88; 54.17 [49.69–57.66]	Reported 12/13; 48.34–63.46; 55.12 [52.80–58.86]
Male, %	Reported 95/95; 27.63–75.00; 55.32 [51.06–60.00]	Reported 82/82; 27.63–75.00; 55.47 [51.06–60.00]	Reported 13/13; 36.36–65.57; 54.84 [50.00–60.00]
Baseline BMI (kg/m²)	Reported 50/95; 22.60–31.09; 26.62 [25.38–28.45]	Reported 46/82; 22.60–31.09; 26.58 [25.38–28.45]	Reported 4/13; 25.73–28.64; 27.39 [26.34–28.34]
T2DM duration (years)	Reported 71/95; 0.79–26.39; 5.66 [4.46–7.11]	Reported 60/82; 1.03–26.39; 5.62 [4.42–7.18]	Reported 11/13; 0.79–10.00; 6.15 [3.20–6.86]
Primary endpoints reported	ALT: 95 (100.0%); TG: 95 (100.0%); HOMA-IR: 60 (63.2%); CAP: 21 (22.1%)	ALT: 82 (100.0%); TG: 82 (100.0%); HOMA-IR: 53 (64.6%); CAP: 20 (24.4%)	ALT: 13 (100.0%); TG: 13 (100.0%); HOMA-IR: 7 (53.8%); CAP: 1 (7.7%)
Additional/robustness endpoints reported	AST: 84 (88.4%); HDL-C: 30 (31.6%); LSM: 12 (12.6%)	AST: 74 (90.2%); HDL-C: 25 (30.5%); LSM: 11 (13.4%)	AST: 10 (76.9%); HDL-C: 5 (38.5%); LSM: 1 (7.7%)
Safety reporting	31 (32.6%)	26 (31.7%)	5 (38.5%)
Risk of bias (RoB 2 overall)	Low: 3 (3.2%); some concerns: 92 (96.8%); high: 0 (0.0%)	Low: 3 (3.7%); some concerns: 79 (96.3%); high: 0 (0.0%)	Low: 0 (0.0%); some concerns: 13 (100.0%); high: 0 (0.0%)

Values are summarized at the trial level. Continuous variables are reported as min–max; median [IQR]. Baseline demographics were summarized descriptively using trial-level central values (average of intervention and comparator arms when both were available); the number of trials reporting each variable is provided. Add-on design denotes TCM added to background Western medicine in both arms (TCM + WM vs. WM). Mixed design denotes non-uniform background therapy between arms. AE, adverse event; US, ultrasound; ALT, alanine aminotransferase; AST, aspartate aminotransferase; TG, triglyceride; HDL-C, high-density lipoprotein cholesterol; HOMA-IR, homeostasis model assessment of insulin resistance; CAP, controlled attenuation parameter; LSM, liver stiffness measurement; WM, Western medicine.

Endpoint means, standard deviations (SD)/standard errors (SE)/95% confidence intervals (95%CI), and units were recorded; data from figures were digitized by two reviewers; time was unified in weeks. Units were retained in their native clinical form (e.g., ALT/AST in U/L; CAP in dB/m), and all syntheses used mean differences (MD) in original units; unit fields and effect-type coding are documented in **Supplementary Material 2**, **Supplementary Table 4** (Unit and EffectType columns). Effect sizes and variances were computed in batches in R under a prespecified rule that TE >0 indicates clinical improvement, mapping diverse endpoints onto a common positive-direction scale. Outcome-specific directionality (lower better vs. higher better) was prespecified based on clinical meaning and documented in **Supplementary Material 2**, **Supplementary Table 4** (Directionality column). Specifically, for LowerBetter outcomes (ALT, AST, TG, HOMA-IR, CAP, LSM), improvement corresponds to a decrease and TE was defined as:


$$TE={\bar{X}}_{control}-{\bar{X}}_{treat}$$

For HigherBetter outcomes (notably HDL-C), improvement corresponds to an increase and TE was defined as:


$$TE={\bar{X}}_{treat}-{\bar{X}}_{control}$$

Mean difference (MD) was used for all outcomes in this review because units were consistent across the included trials; SMD was prespecified only as a contingency when units were not reconcilable but was not required in the final dataset (**Supplementary Material 2**, **Supplementary Table 4**, Effect Type column).

As this was a systematic review, no medicinal materials were procured by us. For each included trial, when reported we extracted (i) product type (classic/custom formula, Chinese patent medicine, single herb/extract), (ii) English and Latin names of herbs, and (iii) manufacturer/supplier, approval or batch number, and quality control information (e.g., marker compounds, assay methods) for Chinese patent medicines or extracts. Where trials did not report such information, items were recorded as NR.

### Knowledge graph construction

Entities (formulas/Chinese patent medicines, herbs, TCM syndromes/symptoms, outcomes, adverse events [AEs]) and relations (formula–herb; formula–syndrome/symptom; formula–outcome; formula/Chinese patent medicine–AE) were derived from the standardized tables. For each formula × outcome pair, aggregated TE and directional consistency informed edge weights/colors; node weights reflected study counts. Multi-arm trials were split by intervention group while retaining trial attributes, including Design_stratum (add-on/mixed) and intervention category metadata. For knowledge-graph visualization, multi-arm trials were represented as separate intervention nodes to preserve intervention-level relations; however, for meta-analytic pooling and meta-regression, shared-control multi-arm structures were handled using prespecified Cochrane-style rules to avoid double-counting (see “Meta-analysis”—handling of multi-arm trials and shared controls). Communities were detected by the Louvain algorithm (labels 1–5 used only for description). Details are provided in **Supplementary Material 2**.

Safety/AEs (coding and completeness): Because safety outcomes are frequently underreported, we explicitly distinguished “AE not reported (NR/unclear)” from “AE not observed (explicitly stated no AEs)” at the trial level. AE reporting status was coded as reported (Y), explicitly stated no AEs (N; zero events observed), or not reported/unclear (NR/unclear) (**Supplementary Material 2**, **Supplementary Table 1**, column BH: AE_reported). AE types were summarized only among trials with available safety information (Y or N), and the reported AE spectrum is tabulated in **Supplementary Material 2** (**Supplementary Table 5**) and visualized in **Supplementary Figure 3**.

### Meta-analysis

From the knowledge graph, we pre-specified primary endpoints to maximize four-domain coverage and cross-trial comparability—ALT (*k* ≈ 70) and TG (*k* ≈ 74) for high evidence density with uniform units, HOMA-IR (*k* ≈ 47 after harmonizing spellings) for the metabolic domain, and CAP (*k* ≈ 8) as the imaging marker—with AST (*k* ≈ 77) retained for robustness. These outcomes were predominantly reported as means ± SD at the most populated 12-week timepoint, permitting MD-based pooling.

To reduce clinical heterogeneity and enhance interpretability, we prespecified a TCM intervention taxonomy using the trial-level variable TCM_category (**Supplementary Material 2**, **Supplementary Table 1**, column AN): classic and custom denote classic and custom formulas, whereas patent and single denote Chinese patent medicines and single-herb preparations/extracts, respectively. Primary metabolic endpoints (ALT, AST, TG, and HOMA-IR) were analyzed at the 12-week timepoint to maximize comparability across trials. Imaging endpoints (CAP and LSM) were synthesized using a broader 8- to 24-week window due to reporting variability; when multiple timepoints were available, we preferentially selected the latest assessment within the prespecified window.

To strengthen the control of confounding from background WM, we conducted prespecified stratified analyses by trial design (add-on vs. mixed; **Supplementary Material 2**, **Supplementary Table 1**, column BA) and explicitly assessed background-therapy comparability for mixed trials (balanced/unclear/imbalanced; **Supplementary Material 2**, **Supplementary Table 1**, column AZ). Accordingly, primary efficacy conclusions are based on add-on trials, while mixed trials are presented as exploratory/sensitivity evidence. In addition, we conducted random-effects meta-analyses stratified by TCM_category (classic/custom/patent/single) and performed sensitivity analyses restricted to formulas only (classic + custom); our main analysis excluded single-herb/extract trials due to sparse evidence and to avoid mixing fundamentally different intervention types, with single-herb/extract trials presented separately as exploratory evidence.

Risk of bias and certainty of evidence: Risk of bias was assessed with the Cochrane RoB 2 tool across five domains (D1: randomization process; D2: deviations from intended interventions; D3: missing outcome data; D4: measurement of the outcome; D5: selection of the reported result) by two independent reviewers with third-party adjudication, and visualized in R (robvis). To ensure transparent and reproducible judgments under common reporting gaps in this literature, we prespecified domain-level decision rules, including explicit handling of missing information (defaulting to “some concerns” when reporting was insufficient to support a “low” judgment; **Supplementary Material 2**, **Supplementary Table 10**). Certainty of evidence for each primary outcome was evaluated using the GRADE approach and summarized in a Summary of Findings table (downgrading for risk of bias, inconsistency, imprecision, and publication bias as applicable; **Supplementary Material 2**, **Supplementary Table 9**).

Handling of multi-arm trials and shared controls: When a trial included multiple eligible TCM arms sharing a single control group, we avoided double-counting the shared control. Following Cochrane guidance, we preferentially combined similar TCM arms within the same TCM_category into a single comparison (pooling means/SDs and sample sizes). If arms could not be meaningfully combined (e.g., belonging to different TCM_category), we split the control group sample size evenly across comparisons for variance estimation while keeping the original control mean and SD unchanged. In all cases, each study contributed at most one independent contrast per TCM_category per outcome–time window to the primary pooling.

For data pooling, a restricted maximum likelihood (REML)-based random-effects model was used. We reported the pooled effect size, 95% CI, *I*² statistic, *τ*², and 95% prediction interval (PI). Prediction intervals were computed for key outcomes to reflect the expected range of effects in a future single study under the random-effects model $\left(\text{μ}\pm t_{k-2,0.975}\times \sqrt{SE_{\text{μ}}^{2}+{\text{τ}}^{2}}\right)$. Prediction intervals were computed for syntheses with sufficient studies (typically *k* ≥ 3). Small-study effects and potential publication bias were assessed using funnel plots. Robustness was examined via Baujat plots, leave-one-out analysis, and top-Δ analysis. The PI results are summarized in **Supplementary Material 2** (**Supplementary Table 7**, **S8**).

### Weighted meta-regression

Separate models were fitted for add-on and mixed designs using the rma.mv function, with random effects specified at the study level (SourceID). Sample size (per arm) and treatment course (weeks) served as core covariates (with splines applied if necessary), while herb dosage and intervention type (TCM_category) were included as optional covariates. To minimize within-study dependence and prevent double-counting from multi-arm/shared-control structures, the meta-regression dataset was constructed such that each SourceID contributed at most one effect size per outcome within the prespecified time window (and, when stratified, per TCM_category). Accordingly, the analytic unit for meta-regression and threshold modeling was effectively study-level. This analysis aimed to identify “design thresholds,” defined as evidence-derived ranges of sample size and duration in which effect estimates show improved stability (i.e., reduced variability) and consistency. Adjusted effect curves, candidate thresholds, and coefficient summaries were outputted.

### Phenotypic clustering and strategy matrix

A binary matrix was constructed based on standardized TCM syndromes/symptoms (reported = 1; unreported [NR] was retained in a separate column). The ConsensusClusterPlus package (Jaccard distance, ward.D2 linkage) was used to evaluate the cumulative distribution function (CDF), Δ-area, and sample tracking plots for *K* = 2–8 to determine the optimal number of clusters.

Cluster profiles and discriminative features were visualized, and mappings among clusters, prescriptions, and outcomes were used to generate a “phenotype × prescription” strategy matrix.

### Machine learning

**Figure 2:**
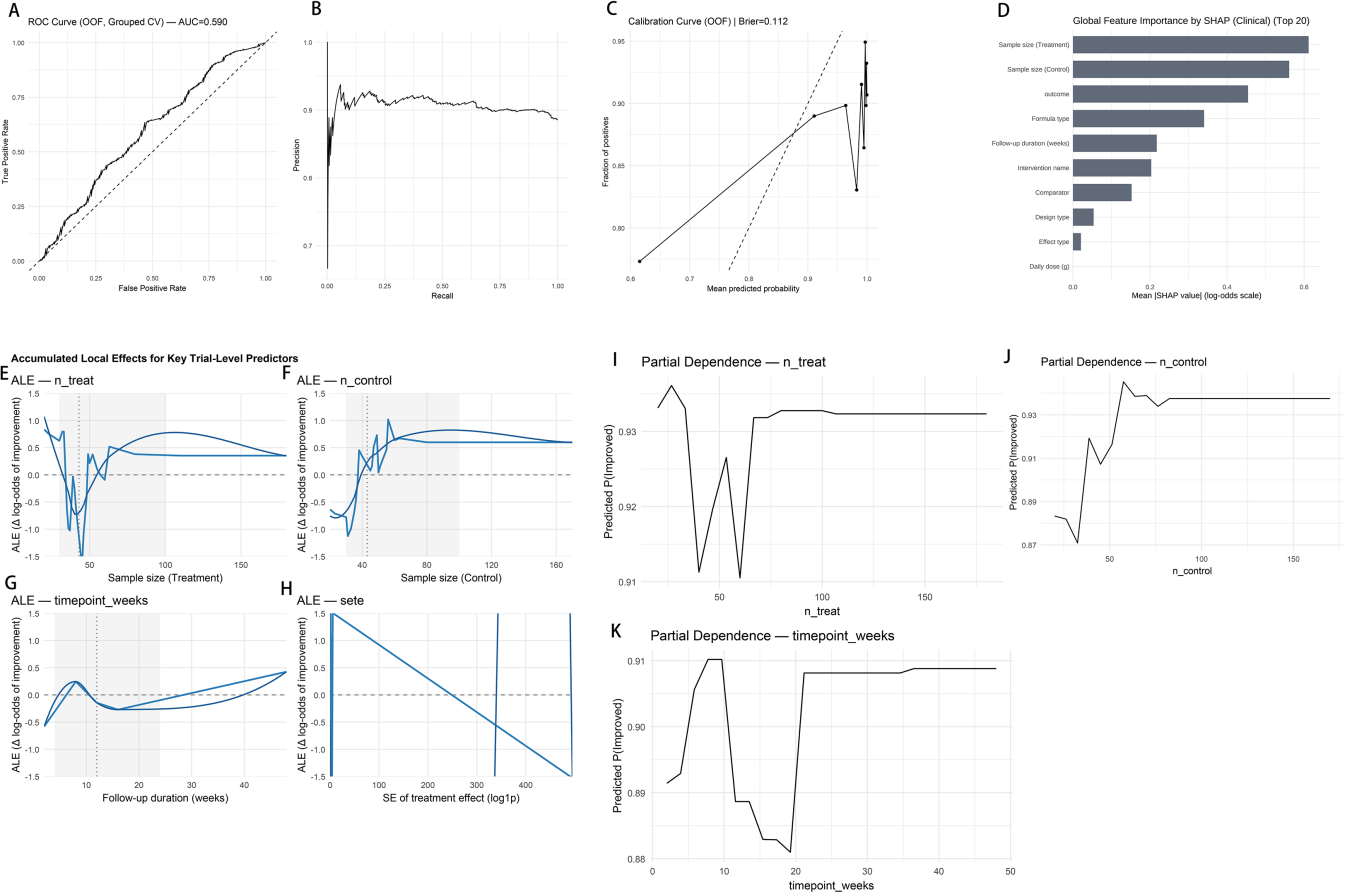
Model performance and interpretability (OOF). **(A)** ROC curves (grouped cross-validation; AUC shown). **(B)** PR curves. **(C)** Calibration (Brier = 0.112). **(D)** Global SHAP (top 20): trial-level variables—*n*_treat_/*n*_control_, follow-up weeks, design type—rank highest. **(E)** ALE for *n*_treat_/*n*_control_, weeks, SE_TE: small-sample amplification → post-threshold stabilization; higher SE_TE is unfavorable. **(F)** PDP (same variables) consistent with ALE.

For classification (TE > 0) and regression (continuous TE) models, each “outcome × trial arm” was treated as an independent sample. XGBoost was used as the primary model, with glmnet and ranger employed for sensitivity analyses. Model performance was evaluated using fivefold study-grouped cross-validation (GroupKFold by SourceID), such that all rows from the same SourceID were assigned to the same fold to prevent leakage; performance metrics were computed from aggregated out-of-fold (OOF) predictions (**Supplementary Material S2**, **Supplementary Table 11**), including ROC-AUC (OOF), PR-AUC (OOF), Brier score (OOF), and calibration diagnostics (e.g., 10-bin ECE; **Supplementary Material S2**, **Supplementary Table 11**; calibration curve in **Figure 2C**). As robustness checks, we conducted a leave-one-study-out (LOSO-like) sensitivity analysis and assessed driver stability (**Supplementary Material S2**, **Supplementary Table 12**) and performed subset robustness analyses restricting to larger trials (e.g., *n* ≥ 40 per arm) or lower-uncertainty effects (e.g., SE_TE ≤ median) (**Supplementary Material S2**, **Supplementary Table 13**). SHAP (SHapley Additive exPlanations) and ALE (accumulated local effects) were applied to characterize the effects of key features and their evidence-derived, hypothesis-generating threshold intervals.

### Statistical software

All analyses were conducted using R 4.4.1. Key R packages included metafor, meta, clubSandwich, dplyr, ggplot2, ConsensusClusterPlus, tidymodels, xgboost, glmnet, ranger, shapviz, pROC, and showtext. Complete analysis scripts are provided in **Supplementary Material 3**.

## Results

### Basic characteristics

We included 95 randomized controlled trials (RCTs; 2015–2025; *n* = 8,813; **Supplementary Material S1**). The intervention arms comprised 4,389 participants, and follow-up duration ranged from 2 to 48 weeks overall (median 12 [12–12]). The trial-level characteristics for all included RCTs (*n* = 95) are presented in **Supplementary Material S2**, and structured datasets supporting the knowledge-graph layers are provided in **Supplementary Material S2** (**Supplementary Tables 1**-**S5**). Detailed trial-level diagnostic criteria/definitions and assessment methods are summarized in **Supplementary Material 2**, **Supplementary Table 1** (columns N–O and BP–BQ).

As summarized in **Table 1**, most trials used an add-on design (*n* = 82; 7,266 participants), with the remainder classified as mixed (*n* = 13; 1,547 participants). The sample size per arm ranged from 20 to 180 (median 40 [33–54]). Trials were predominantly conducted in China (93/95, 97.9%), and diagnosis/ascertainment was guideline-based in all studies (95/95, 100%). Ascertainment (diagnostic) imaging modalities were dominated by ultrasound (75/95, 78.9%). CAP-based assessment was used as an ascertainment modality in 10 trials (10.5%), while other modalities were less frequent (liver CT 6/95, 6.3%; LSM 2/95, 2.1%; MRI-PDFF 1/95, 1.1%; and ¹H-MRS 1/95, 1.1%). Background therapy was reported in 91 trials (95.8%). Most comparators were Western medicine/standard care (87/95, 91.6%), with fewer placebo-controlled trials (3/95, 3.2%).

Interventions were mainly TCM compound formulas (74/95, 77.9%; 25 classical and 49 custom), followed by Chinese patent medicines (17/95, 17.9%) and single herbs (4/95, 4.2%). Syndrome/pattern information was reported in 60 trials (63.2%), whereas 32 trials (33.7%) did not report or were unclear/NR. Baseline demographics were variably reported at the trial level: age was available in 89/95 trials (median 54.18 years [49.72–57.66]), male proportion in 95/95 (median 55.32% [51.06–60.00]), BMI in 50/95 (median 26.62 kg/m² [25.38–28.45]), and T2DM duration in 71/95 (median 5.66 years [4.46–7.11]).

Regarding outcome coverage, ALT and TG were reported in all trials (95/95, 100%), whereas HOMA-IR (60/95, 63.2%) and CAP as an outcome endpoint (21/95, 22.1%) were less frequently reported. Notably, CAP may appear either as an ascertainment modality or as a follow-up endpoint; this proportion here refers specifically to CAP reported as an outcome and is not intended to match ascertainment-modality reporting. Among additional/robustness endpoints, AST was reported in 84 trials (88.4%), HDL-C in 30 (31.6%), and LSM in 12 (12.6%).

**Figure 3:**
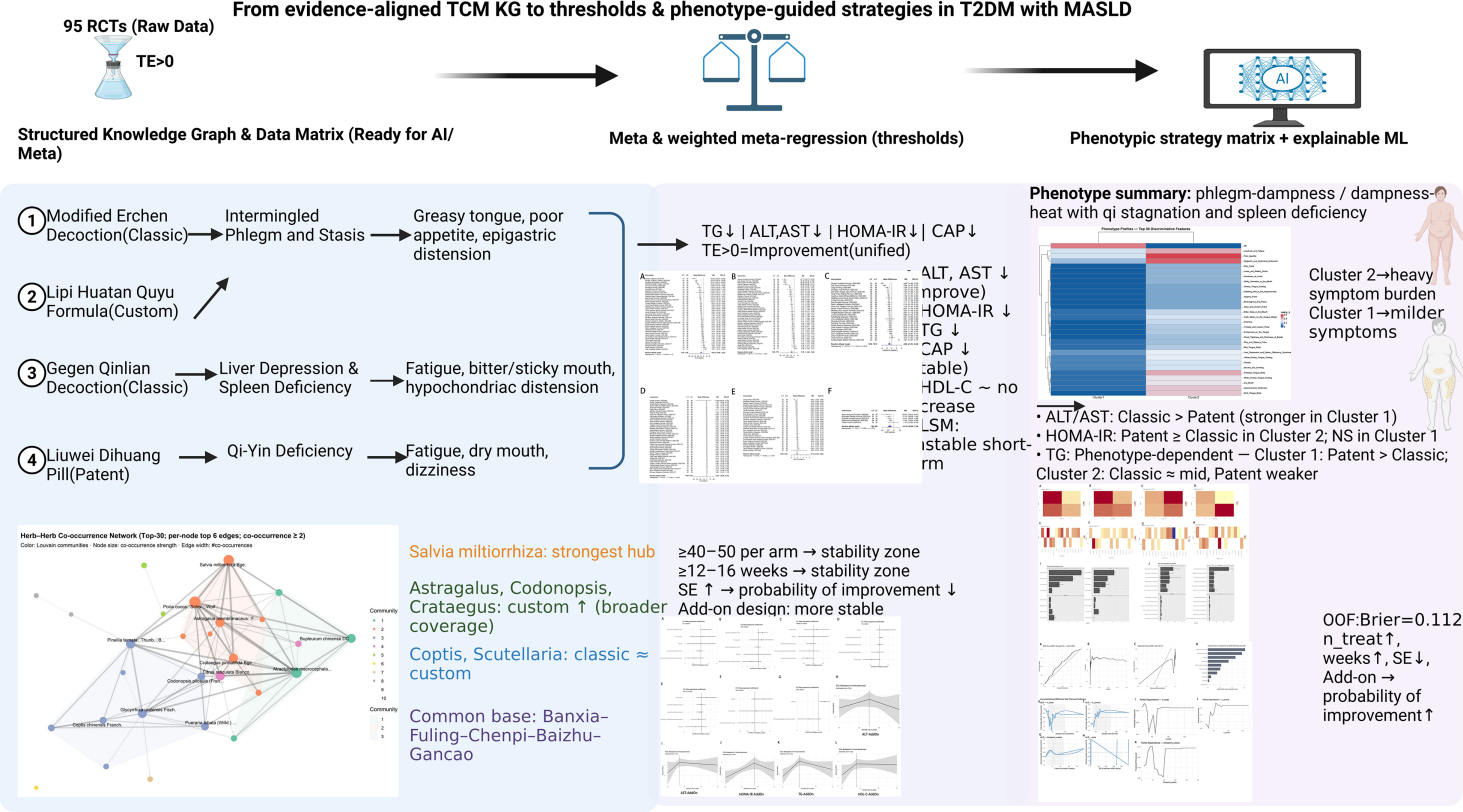
Evidence-to-translation roadmap. The diagram integrates the study workflow into four key domains: evidence pipeline: standardization of 95 RCTs (TE > 0) into a structured knowledge graph; unified outcome fingerprint: visual summary of consistent improvements in ALT, AST, HOMA-IR, TG, and CAP (with short-term LSM noted as unstable); design thresholds: empirically derived sample size (≥40–50/arm) and duration (≥12–16 weeks) benchmarks for reproducibility; and strategy matrix: phenotype-stratified prescribing implications (e.g., classical formulas for mild profiles vs. custom formulas for phlegm-damp/heat).

Safety reporting was limited: 31 trials (32.6%) reported AE data and 10 (10.5%) explicitly stated that no AEs occurred (zero events observed), while 54 (56.8%) did not report safety outcomes (NR/unclear) (**Supplementary Material 2**, **Supplementary Table 1**, column BH; **Supplementary Figure 1**). RoB 2 overall ratings were predominantly “some concerns” (92/95, 96.8%), with three trials rated “low” risk and none rated “high” (**Supplementary Figure 1**). **Figure 3** provides an at-a-glance roadmap for readers (including non-TCM practitioners), summarizing our end-to-end workflow and key outputs—from evidence standardization and knowledge-graph organization to meta-analytic synthesis, design thresholds, and phenotype-guided strategy implications.

### Knowledge graph construction

**Figure 4:**
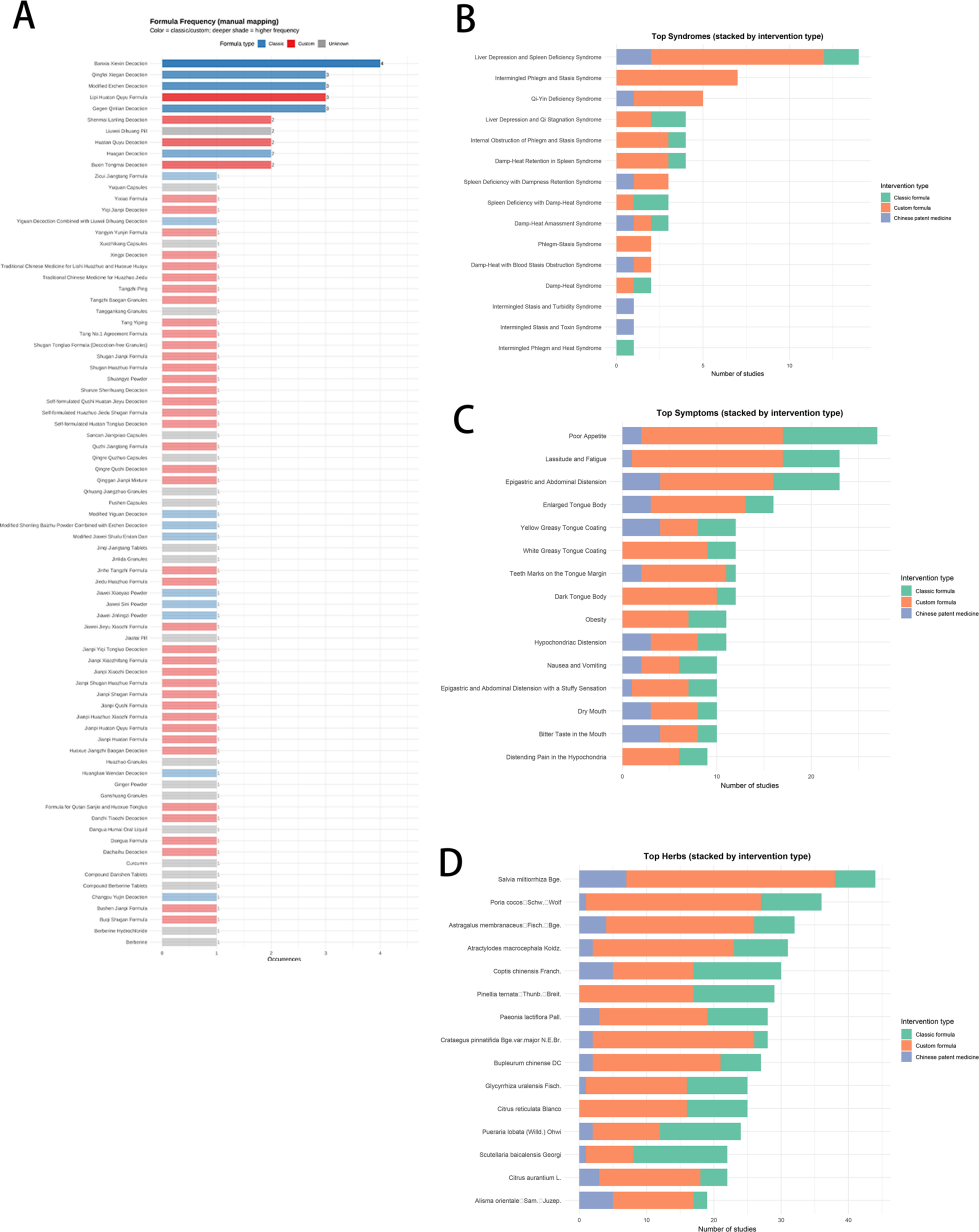
Distribution of interventions, syndromes, symptoms, and herbs (by number of studies). **(A)** Formula frequency by type. **(B)** Top 15 TCM syndromes. **(C)** Top 15 TCM symptoms. **(D)** Top 15 herbs. Bars are stacked by intervention type.

Interventions–syndromes/symptoms–core herbs (**Figure 4**) and TCM intervention distribution (**Figure 4A**): Classical formulas were most commonly used overall. Banxia Xiexin Decoction appeared in four trials, Qingfei Xiegan Decoction, Modified Erchen Decoction, and Gegen Qinlian Decoction in three trials each, and Huagan Decoction in two. Among custom formulas, Lipi Huatan Quyu appeared in three trials, and Shenmai Lanling Decoction, Huatan Quyu Decoction, and Buxin Tongmai Decoction in two each. For Chinese patent medicines, Liuwei Dihuang Pill appeared in two trials. All remaining TCM interventions each appeared once.

Top syndromes (**Figure 4B**): The leading syndromes were liver depression and spleen deficiency, intermingled phlegm–stasis, and Qi–Yin deficiency. By category, custom formulas contributed the largest share, followed by classical formulas, with CPMs the least.

Top symptoms (**Figure 4C**): Poor appetite, lassitude, and epigastric distension were most common; classical formulas covered the broadest symptom spectrum, then custom formulas, with CPMs the lowest.

Top herbs (**Figure 4D**): *Salvia miltiorrhiza* ranked first, followed by *Poria*, *Astragalus membranaceus*, and *Atractylodes macrocephala*. Herb contributions were highest in custom, then classical formulas, and lowest in CPMs.

**Figure 5:**
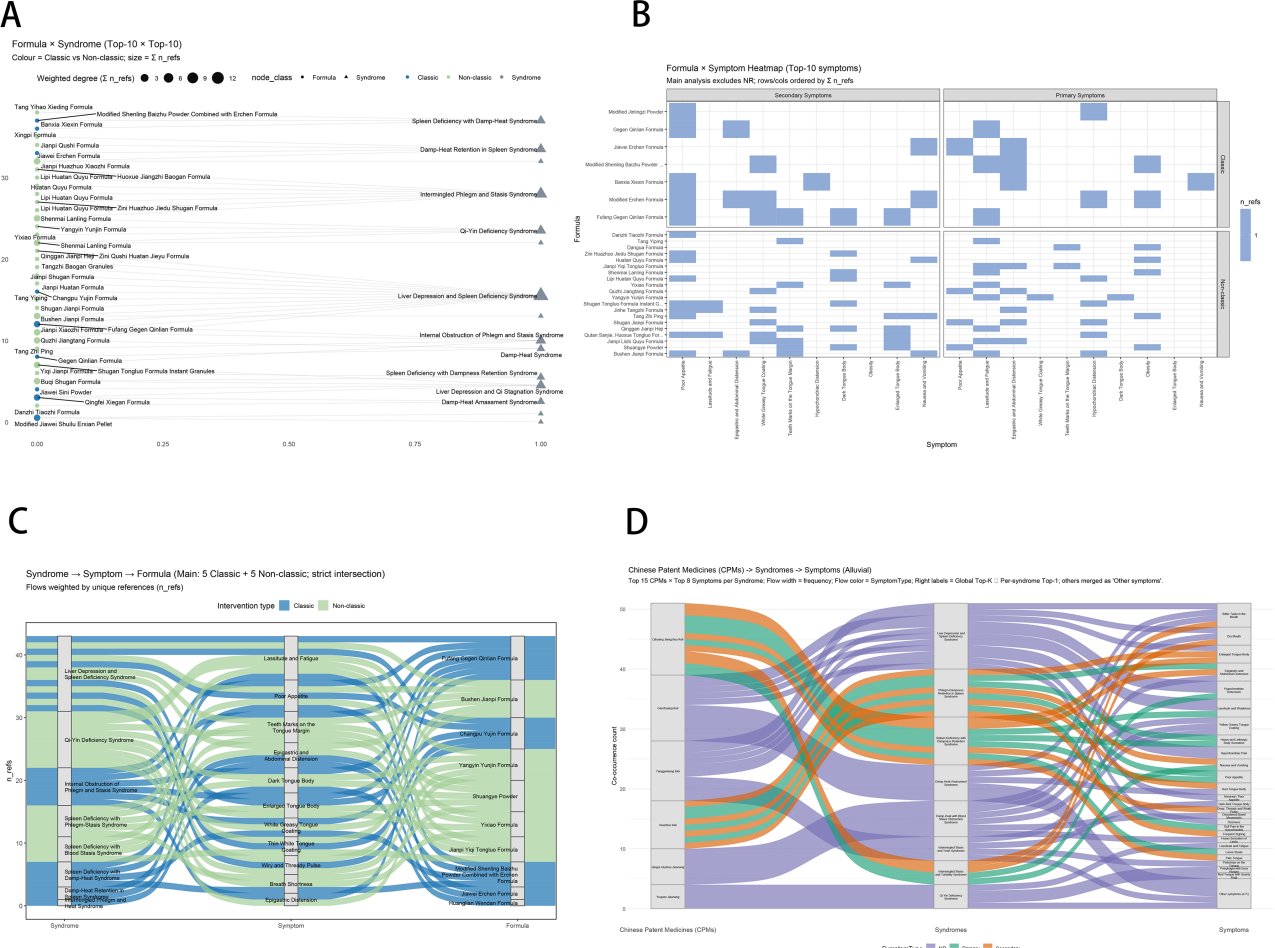
Formula–phenotype associations. **(A)** Bipartite network of top 10 formulas × top 10 syndromes. **(B)** Formula × top 10 symptoms heatmap (primary vs. secondary). **(C)** Sankey: syndrome → key symptom → formula (five classical + five custom). **(D)** Alluvial: Chinese patent medicine → syndrome → symptom.

Interventions–syndrome/symptom associations (**Figure 5**)—formula × syndrome network (**Figure 5A**). Links were densest for liver depression and spleen deficiency, intermingled phlegm–stasis, and Qi–Yin deficiency. Custom formulas (e.g., Lipi Huatan Quyu, Shenmai Lanling) predominated, with classical formulas (e.g., Banxia Xiexin, Gegen Qinlian) close behind.

Formula–symptom heatmap (**Figure 5B**): Primary symptoms clustered as poor appetite, lassitude/fatigue, abdominal distension, and white greasy tongue coating, with classical formulas showing the most concentrated pairings—Banxia Xiexin Decoction and Gegen Qinlian Decoction in particular. Secondary symptoms—tooth-marked tongue and abdominal bloating—were more extensively covered by custom formulas, notably Lipi Huatan Quyu Formula and Shenmai Lanling Formula.

Sankey diagram (**Figure 5C**): The primary syndrome → key symptom → formula pathway concentrated on liver depression and spleen deficiency, Qi–Yin deficiency, and intermingled phlegm–stasis. Key symptoms were lassitude/fatigue, poor appetite, tooth-marked tongue, and abdominal distension. Highly connected formulas included classical regimens—Compound Gegen Qinlian Formula, Modified Erchen Decoction, Shenling Baizhu Powder plus Erchen Decoction—and custom regimens—Bushen Jianpi Formula, Yangyin Yunjin Formula, Yixiao Formula.​ CPM → syndrome → symptom (**Figure 5D**). CPMs such as Qinggan Jiangzhuo, Ganhuang, and Tanggankang mainly targeted liver depression and spleen deficiency, spleen deficiency with dampness, or damp heat, corresponding to lassitude/fatigue, poor appetite, and epigastric distension.

**Figure 6:**
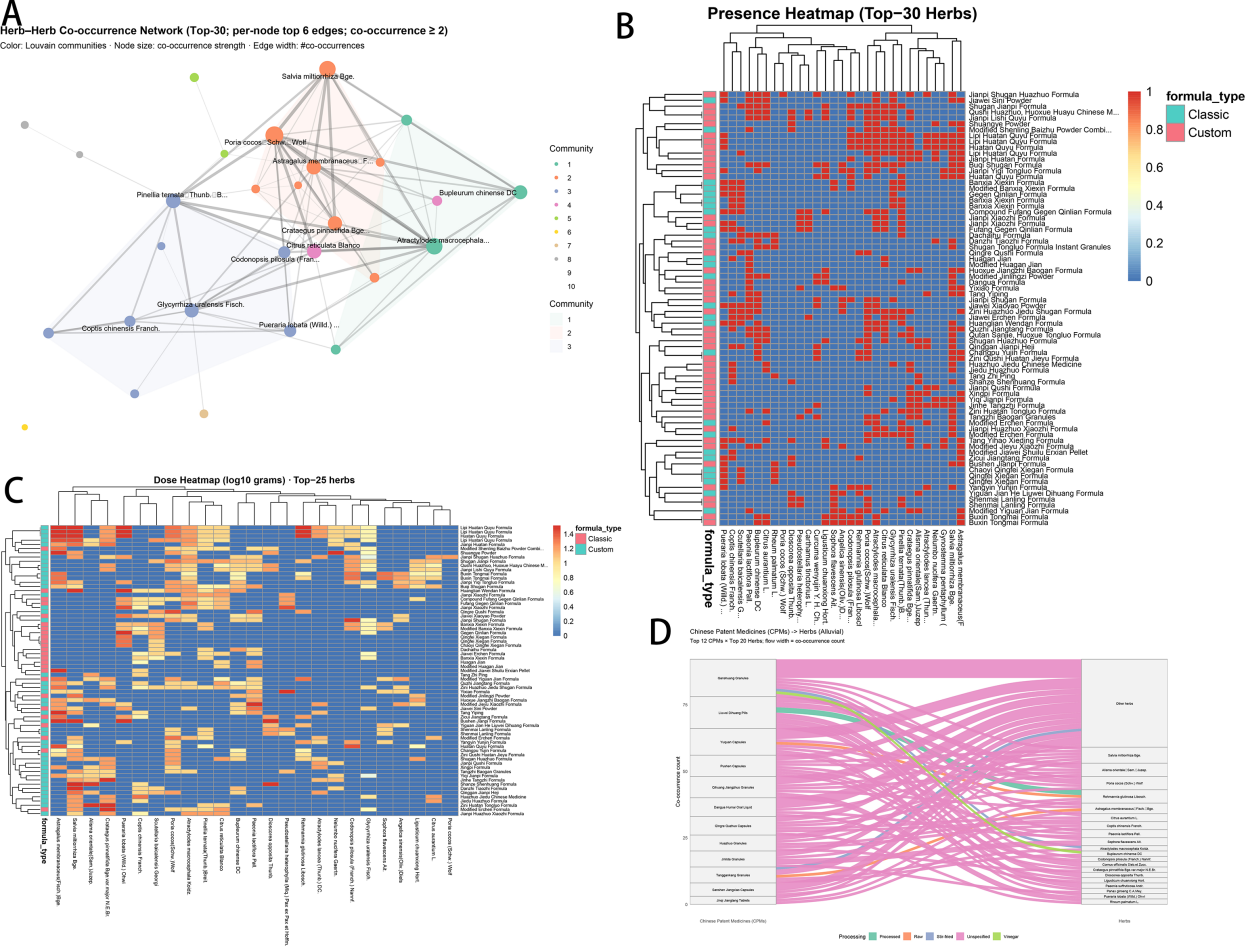
Herb co-occurrence, dosage, and processing. **(A)** Herb–herb co-occurrence network with Louvain communities. **(B)** Presence/absence heatmap (representative formulas × top 30 herbs). **(C)** Dosage heatmap (top 25 herbs; log10 grams). **(D)** Patent medicine → herb Sankey.

Herb co-occurrence, dosage, and processing (**Figure 6**)—herb–herb network (**Figure 6A**): *Salvia miltiorrhiza* acted as the strongest hub, forming three clusters: cluster 1: *Salvia miltiorrhiza*—*Astragalus membranaceus*—*Crataegus pinnatifida* var. *major*/*Citrus reticulata*—*Atractylodes macrocephala*; cluster 2: *Pinellia ternata*—*Glycyrrhiza uralensis*—*Coptis chinensis*—*Codonopsis pilosula*/*Pueraria lobata*; side cluster: including *Bupleurum chinense*, connected to *Atractylodes macrocephala*.

Top herbs and modules (**Figure 6B**): Classical and custom formulas shared a universal base (*Pinellia*–*Poria*–*Citrus reticulata*–*Atractylodes macrocephala*–*Glycyrrhiza*), commonly augmented by selective pairs (*Astragalus* and *Codonopsis*; *Coptis* and *Scutellaria*) and *Bupleurum*.

Dosage heatmap (**Figure 6C**): A moderate-dose base (e.g., *Poria*, *Pinellia*, *Citrus reticulata*) was superimposed with high-dose hotspots (e.g., *Salvia*, *Bupleurum*, *Crataegus*).

CPM–herb flow diagram (**Figure 6D**): Various Chinese patent medicines, represented by Ganshuang Granule and Liuwei Dihuang Pill, were mainly connected to core herbs such as *Salvia miltiorrhiza*, *Alisma orientale*, and *Poria cocos*.

**Figure 7:**
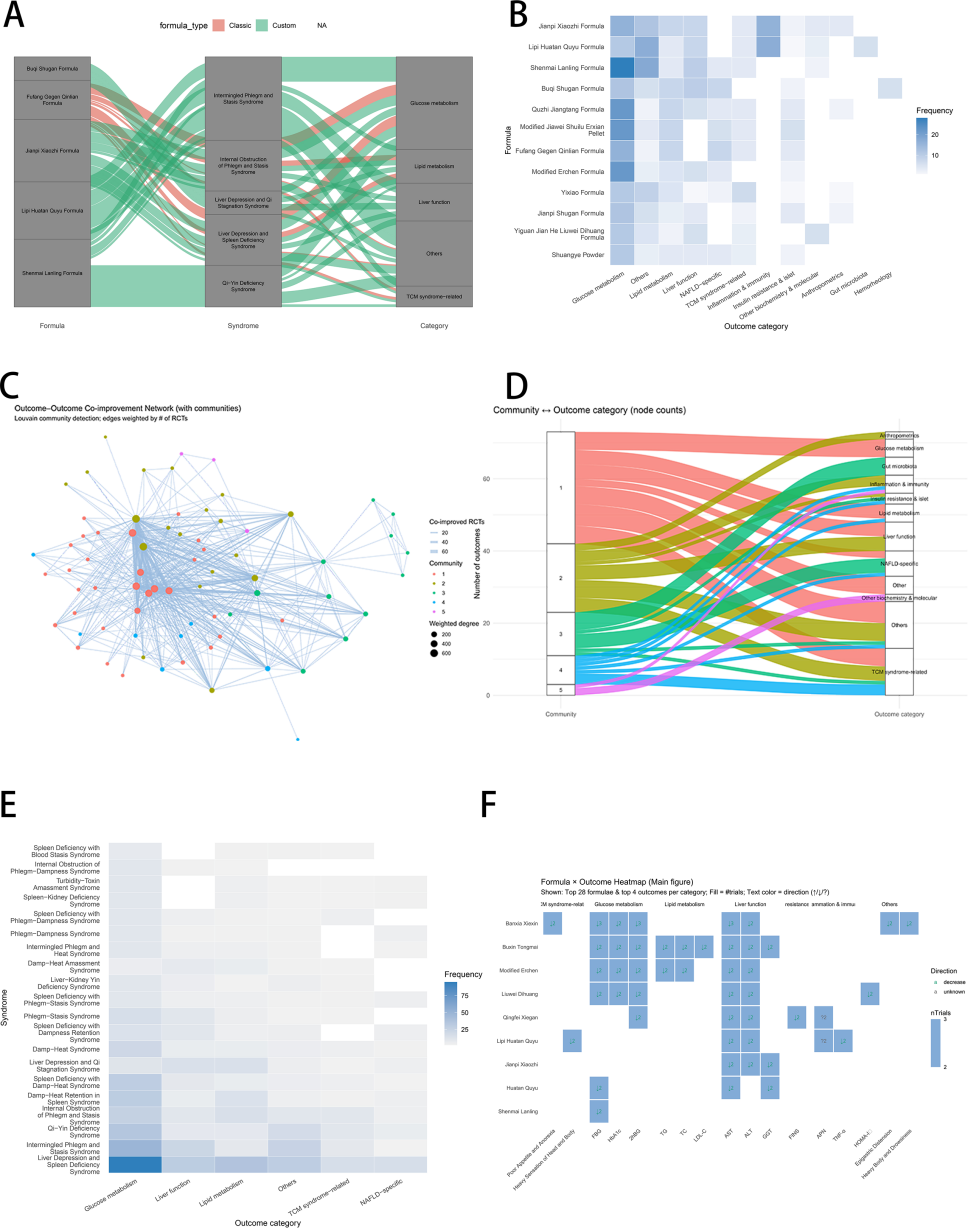
Formula–syndrome pathways to outcomes. **(A)** Sankey: formula → syndrome → outcome. **(B)** Heatmap: formula × outcome domains. **(C)** Outcome network modules. **(D)** Community–outcome mapping. **(E)** Heatmap: high-frequency syndromes × outcomes. **(F)** Heatmap: formulas × specific outcome indicators. ALT, alanine aminotransferase; AST, aspartate aminotransferase; HOMA-IR, Homeostatic Model Assessment of Insulin Resistance; TG, triglycerides; CAP, controlled attenuation parameter; LSM, liver stiffness measurement.

Formula–syndrome pathways to outcomes (**Figure 7**)—main pathway (**Figure 7A**): Formulas such as Buqi Shugan, Compound Gegen Qinlian, Jianpi Xiaozhi, Lipi Huatan Quyu, and Shenmai Lanling connected to outcomes through intermingled phlegm–stasis, liver depression and Qi stagnation, liver depression, and spleen deficiency, and Qi–Yin deficiency. Custom formulas tended to map to glucose/lipid metabolism, whereas classical formulas were relatively more oriented to liver function.

Coverage heatmap (**Figure 7B**): Shenmai Lanling Formula had the widest coverage across outcomes, especially for glucose metabolism and liver function indicators. Jianpi Xiaozhi Formula, Modified Erchen Formula, and Quzhi Jiangtang The formula mainly focused on glucose and lipid outcomes. Fuzheng Shugan Formula and Compound Gegen Qinlian Formula showed a more balanced distribution, covering liver function, inflammatory immunity, and MASLD-specific indicators.

Outcome–outcome network (**Figure 7C**): Hubs (ALT/AST, fasting blood glucose (FBG)/hemoglobin A1c(HbA1c), and total cholesterol (TC)/TG) formed modules of liver function, lipids, glycemia/islet, and inflammation/syndromes. The strongest co-improvements were glucose ↔ lipids and liver function ↔ inflammation, indicating a multidimensional benefit.

Community–outcome mapping (**Figure 7D**): Louvain-detected communities were mapped as follows: community 1: anthropometry, glucose metabolism, liver function; community 2: lipid metabolism, inflammatory immunity; community 3: MASLD-specific indicators and intestinal flora; communities 4–5: molecular biochemistry and TCM-syndrome-related outcomes.

Syndrome × outcome (**Figure 7E**): Liver depression and spleen deficiency syndrome and intermingled phlegm and stasis syndrome were most frequent, corresponding mainly to glucose metabolism, liver function, and lipid metabolism outcomes.

Formula×outcome (**Figure 7F**; **Supplementary Figure 2**): TCM interventions such as Gegen Qinlian Decoction, Modified Erchen Decoction, Huatan Quyu Formula, and Liuwei Dihuang Pill reduced FBG, HbA1c, 2-h blood glucose (2hBG), TG, TC, low-density lipoprotein cholesterol (LDL-C), ALT, AST, gamma-glutamyl transferase (GGT), and CAP in ≥2 randomized controlled trials. Supplementary figures showed concentrated dark blocks for classical formulas in glucose control, lipid lowering, and hepatoprotection, whereas custom formulas displayed stronger intensity for insulin resistance/islet function, inflammation/immunity, anthropometry, gut microbiota, and TCM syndrome outcomes.

Safety (reporting completeness and AE spectrum): Safety reporting was incomplete across the included evidence: 31/95 trials (32.6%) explicitly reported AE data, 10/95 trials (10.5%) explicitly stated that no AEs occurred (zero events observed), whereas 54/95 trials (56.8%) did not report safety outcomes (NR/unclear) (**Supplementary Material 2**, **Supplementary Table 1**, column BH). Accordingly, the AE spectrum is summarized only within trials with available safety information. The reported AEs were mainly mild gastrointestinal and systemic symptoms (e.g., diarrhea, hypoglycemia, gastrointestinal reactions, nausea/vomiting, dizziness, fatigue), with broadly similar distributions across intervention types (**Supplementary Figure 3**; **Supplementary Material 2**, **Supplementary Table 5**).

### Meta-analysis

Heterogeneity and risk-of-bias overview: Across primary biochemical outcomes, heterogeneity was substantial (ALT *I*² ≈ 98%, AST *I*² ≈ 96%, HOMA-IR *I*² ≈ 98%, TG *I*² ≈ 92%), whereas CAP showed low heterogeneity (*I*² ≈ 6%) and LSM showed moderate heterogeneity (*I*² ≈ 64%). Accordingly, we applied REML-based random-effects models and reported τ², 95% prediction intervals (PI), and the *p*-value for heterogeneity (*Q* test) for all syntheses. We further explored heterogeneity via prespecified design stratification (add-on vs. mixed) and robustness diagnostics, including funnel plots, Baujat plots, leave-one-out, and top-Δ analyses (**Supplementary Figures 4**-**S8**). Intervention-type stratification using the prespecified TCM category (classic/custom/patent/single) showed that the pooled effects for the 12-week biochemical endpoints (ALT/AST/TG/HOMA-IR) remained directionally favorable across classic formulas, custom formulas, and patent medicines, although substantial heterogeneity persisted within strata (**Supplementary Material 2**, **Supplementary Table 6**). Evidence for single-herb/extract interventions was sparse (often *k* = 1) and is therefore presented as exploratory; sensitivity analyses restricted to formulas (classic+custom) and excluding single-herb/extract (main) were consistent with the primary conclusions (**Supplementary Material 2**, **Supplementary Table 6**).

Risk of bias and certainty of evidence: Using RoB 2 with prespecified decision rules (**Supplementary Material 2**, **Supplementary Table 10**), overall risk of bias was predominantly rated as “low” or “some concerns,” with concerns most frequently arising from deviations from intended interventions (D2) and missing outcome data (D3); no studies were judged as having an overall “high” risk of bias (**Supplementary Figure 1**; domain-level judgments for each trial are provided in **Supplementary Material 2**, **Supplementary Table 1**, columns BI–BO). Certainty of evidence assessed with GRADE for the primary outcomes is summarized in the Summary of Findings table (**Supplementary Material 2**, **Supplementary Table 9**). Given substantial between-study heterogeneity and, for several biochemical endpoints, wide prediction intervals, certainty was commonly downgraded for inconsistency and/or imprecision, and clinical inferences were framed accordingly.

**Figure 8:**
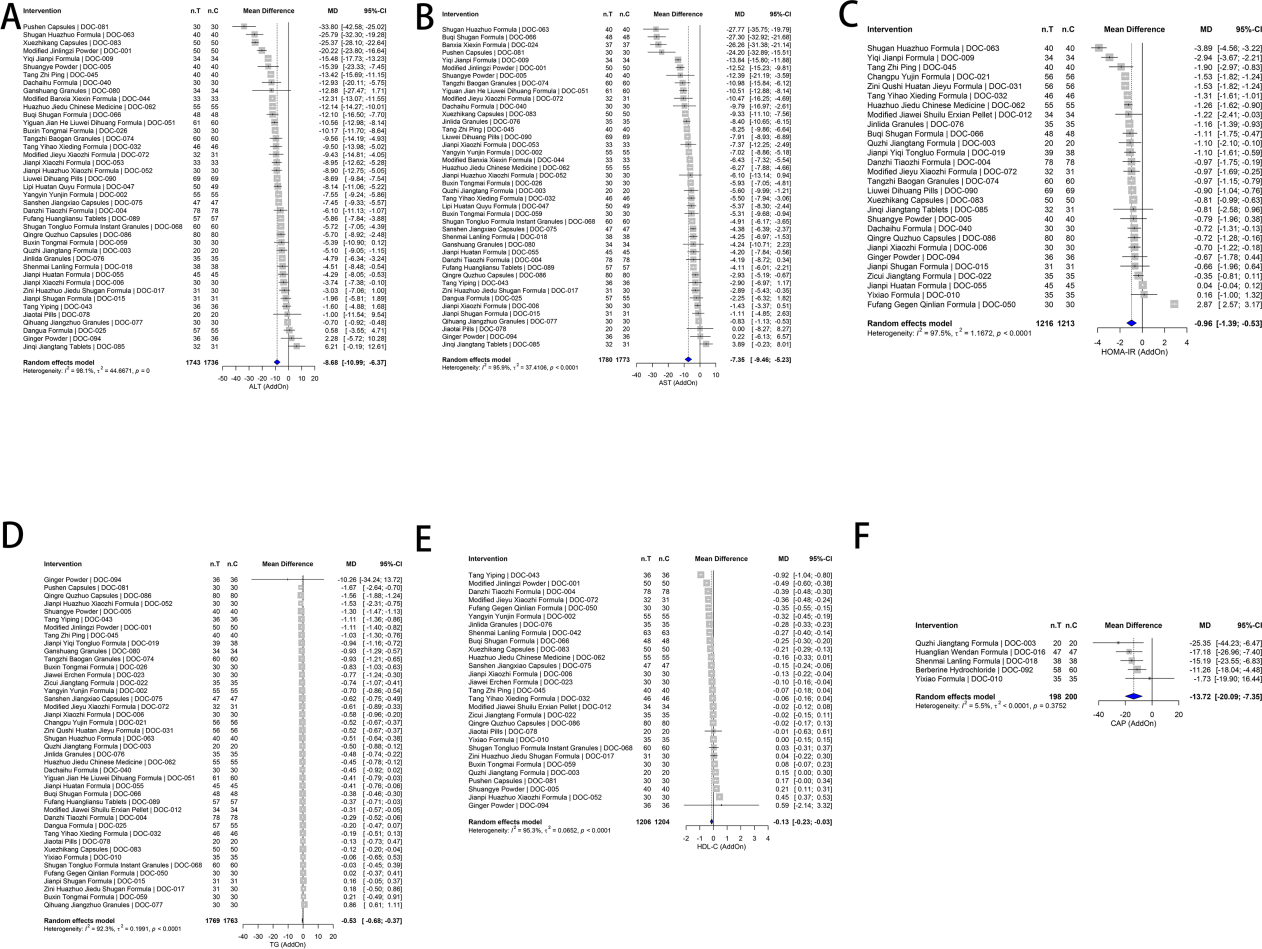
Random-effects meta-analysis in add-on trials. **(A)** ALT, **(B)** AST, **(C)** HOMA-IR, **(D)** TG, **(E)** HDL-C, **(F)** CAP. Effect size is mean difference (MD). Directionality (clinical improvement): MD < 0 for ALT/AST/HOMA-IR/TG/CAP and MD > 0 for HDL-C (HigherBetter). Squares indicate study-specific effects (size proportional to inverse-variance weight), horizontal lines show 95% CI, and diamonds denote pooled effects. *τ*², *I*², and *Q*-test *p*-values are reported in each panel. For cross-outcome comparability, outcome-specific directionality and TE harmonization (TE > 0 = improvement) are prespecified in **Supplementary Table 4**. Additional diagnostics (mixed set; leave-one-out/Baujat; funnel/Egger) are shown in **Supplementary Figures 9**-**S13**.

Add-on therapy (primary efficacy analysis; **Figure 8**): Given balanced background WM regimens by design, the add-on stratum served as the primary basis for efficacy conclusions. In this framework, TCM showed directionally favorable pooled effects for liver enzymes, insulin resistance, and triglycerides. ALT decreased (**Figure 8A**; MD −8.68, 95% CI −10.99 to −6.37; I²=98.1) and AST decreased (**Figure 8B**; MD −7.35, 95% CI −9.46 to −5.23; *I*² = 95.9). HOMA-IR decreased (**Figure 8C**; MD −0.96, 95% CI −1.39 to −0.53; *I*² = 97.5), and TG decreased (**Figure 8D**; MD −0.53, 95% CI −0.68 to −0.37; *I*² = 92.3). However, the corresponding 95% PIs for ALT, AST, HOMA-IR, and TG crossed the null (**Supplementary Material S2**, **Supplementary Tables 7**, **S8**), indicating that the effect size may vary materially across settings and that a future single trial could plausibly observe attenuated or null effects despite directionally favorable pooled means. HDL-C showed a small decrease, which was unfavorable (**Figure 8E**; MD −0.13, 95% CI −0.23 to −0.03; *I*² = 95.3).

CAP decreased with low heterogeneity (**Figure 8F**; MD −13.72 dB/m, 95% CI −20.09 to −7.35; *I*² = 5.5), indicating a comparatively more reproducible short-term imaging signal. Consistently, CAP showed a 95% PI that did not cross the null (**Supplementary Material S2**, **Supplementary Table 7**), supporting a comparatively more reproducible short-term imaging signal. LSM did not show a significant pooled change (**Supplementary Figure 4F**; MD −0.63, 95% CI −1.57 to 0.31), with moderate heterogeneity (*I*² = 64.3%, *τ*²= 0.3472; *p* for heterogeneity = 0.0242); its 95% PI crossed the null (**Supplementary Material S2**, **Supplementary Table 7**), consistent with study-to-study variability and an unstable short-term stiffness signal.

Mixed therapy (Exploratory analysis; **Supplementary Figure 4**): Results from mixed-design trials are presented as exploratory findings because background WM regimens may differ between arms or are incompletely reported. Background-therapy comparability for mixed trials was coded as balanced/unclear/imbalanced and is provided in **Supplementary Material 2** (**Supplementary Table 1**, column AZ: WM_comparability). Under this framework, ALT, AST, HOMA-IR, and TG showed directions consistent with the add-on analyses, but with smaller magnitudes and persistent heterogeneity; prediction intervals were generally wide and frequently crossed the null (**Supplementary Material S2**, **Supplementary Table 8**), reinforcing cautious interpretation.

Bias and robustness: Funnel plots (**Supplementary Figures 4G–K**) were broadly symmetric for ALT, AST, HOMA-IR, TG; HDL-C showed asymmetry (possible small-study effects/heterogeneity). The Baujat plots (**Supplementary Figures 5**, **S6**) indicated that few studies drove Q for ALT/AST/HOMA-IR/TG, and removing them did not change the pooled conclusions; HDL-C showed clustered high-impact points near zero; CAP contributed little and was robust; LSM was study-sensitive and overall non-significant. Leave-one-out and top-Δ analyses (**Supplementary Figures 7**, **S8**) supported directionally favorable pooled effects for ALT, AST, HOMA-IR, TG, and CAP in the add-on primary set; HDL-C remained non-favorable, and the short-term LSM signal remained variable. In the mixed sets, TG showed the most reproducible pooled signal, whereas ALT/AST/HOMA-IR kept direction with attenuated size; HDL-C was uncertain; LSM was unstable.

### Meta-regression

**Figure 9:**
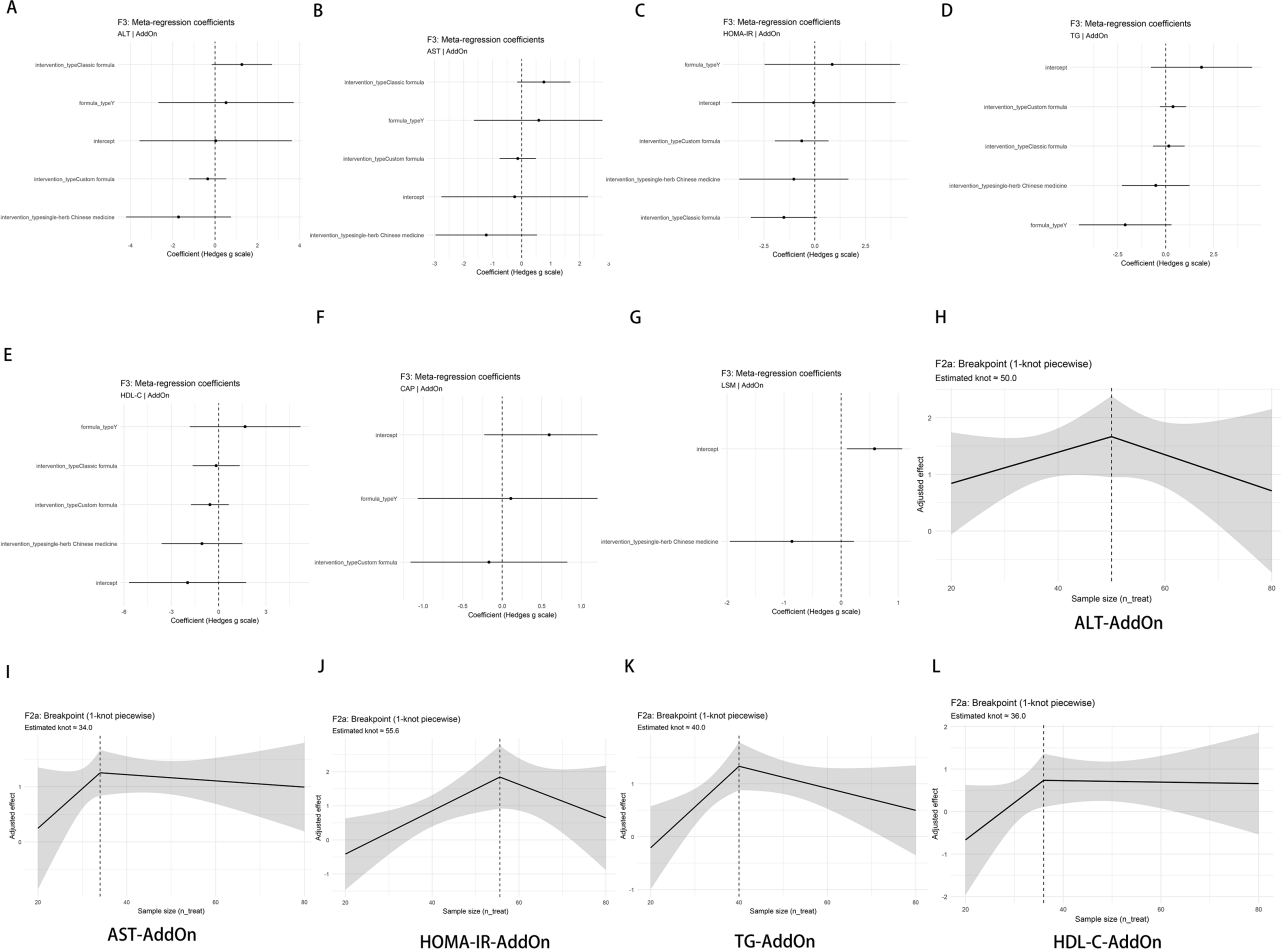
Meta-regression and breakpoint analysis (add-on). **(A–G)** Coefficient forests (REML, Knapp–Hartung): *x*-axis = regression coefficient, points = estimate, lines = 95% CI; covariates per method. Direction follows that of **Figure 7** (ALT/AST/HOMA-IR/TG/CAP/LSM: coef.<0 = greater improvement; HDL-C: coef.>0 = greater improvement). **(H–L)** Piecewise regression (1-knot) for *n*_treat_: solid = adjusted mean effect; shaded = 95% CI; dashed = estimated breakpoint. Model specification and sensitivity checks are shown in **Supplementary Figure 14**.

Most covariate coefficients had 95% CIs crossing zero, indicating no reproducible moderators and aligning with leave-one-out/Baujat findings. ALT, AST, HOMA-IR, TG (**Figures 9A–D**): Improvements were consistent across intervention categories, but moderator effects rarely reached significance. HDL-C (**Figure 9E**): Coefficients fluctuated around zero without reproducible favorable moderation. CAP (**Figure 9F**): Covariate effects were near zero, supporting CAP as the most robust short-term indicator. LSM (**Figure 9G**): Directions were inconsistent with wide intervals, suggesting short-term sensitivity to study-level variation. Sample-size thresholds (add-on; one-knot piecewise on n_treat; **Figures 9H–L**). Approximate inflection points (participants per arm): ALT ~50, AST ~34, HOMA-IR ~56, TG ~40, HDL-C ~36. Effects tended to be amplified in smaller samples and stabilized/declined near these thresholds. We therefore recommend ≥40–50 participants per arm to improve reproducibility and to mitigate small-study effects.

We further reported coefficient forest plots for the mixed set and smoothed marginal-effect curves of n_treat under add-on therapy (**Supplementary Figure 9**). Most coefficients fluctuated around zero with directions consistent with the main add-on regression and no stable significant moderators. The n_treat curves reproduced the “small-sample amplification → post-threshold stabilization/decline” pattern; the HDL-C curve was nearly flat. After covariate adjustment, improvements in ALT/AST/HOMA-IR/TG and the stable CAP reduction remained; HDL-C showed no clear benefit, and LSM remained unstable. The sample-size thresholds provide a design benchmark (≥40–50 per group) for future studies and interpretation.

### Phenotype clustering and stability assessment

**Figure 10:**
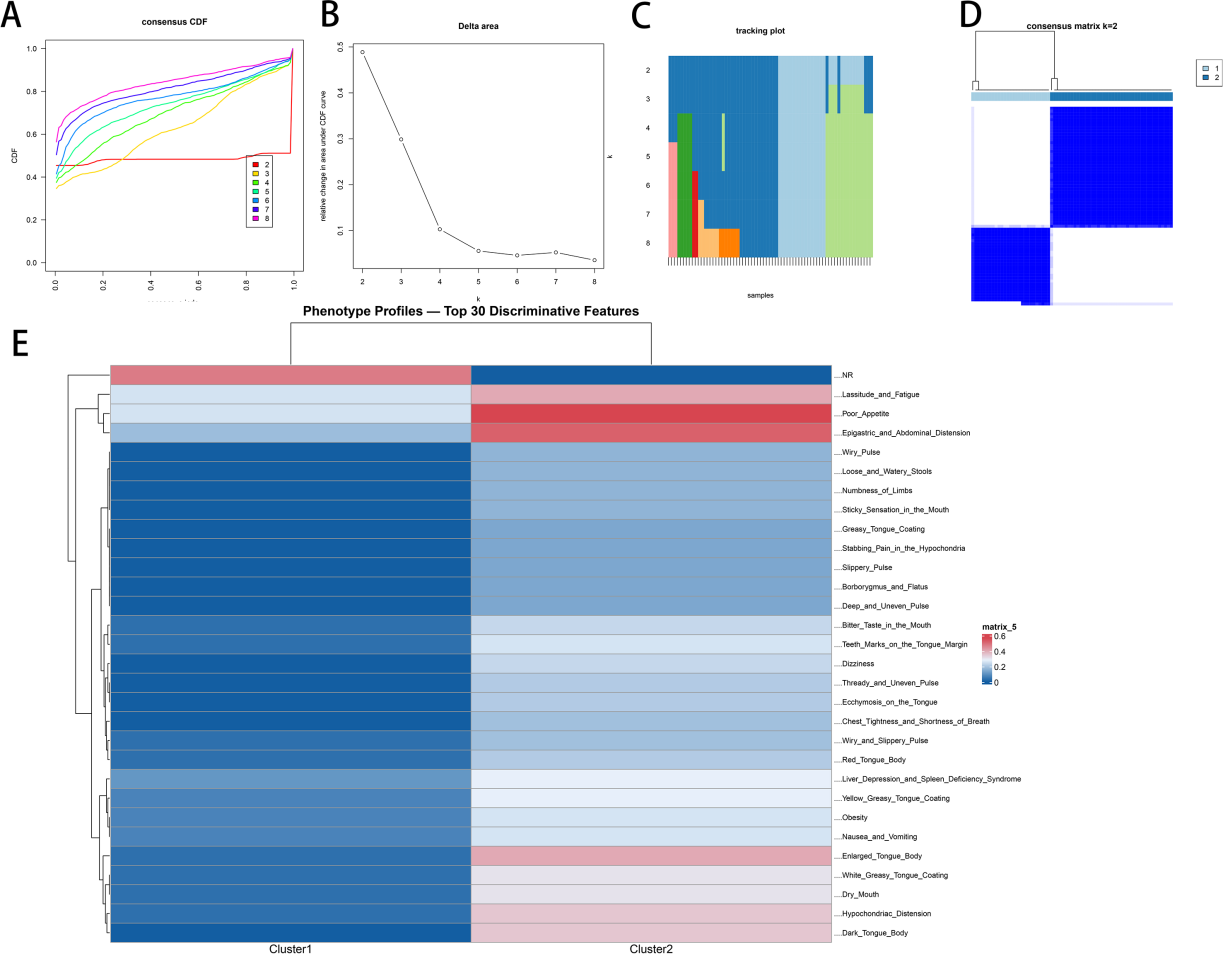
Phenotype clustering and profiling. **(A)** Consensus CDF, **(B)** Δ-area curves, **(C)** tracking plot across *k*, **(D)** consensus matrix at *k* = 2, **(E)** top 30 discriminative-feature heatmap. *k* = 2 yielded the largest stability gain and two high-consistency phenotypes.

Consensus clustering (**Figures 10A–D**; **Supplementary Figure 10**): Clustering was performed on symptoms, tongue–pulse signs, and syndrome labels. The consensus CDF/Δ-area curves showed the largest gain at *k* = 2, after which gains plateaued; tracking plots indicated minimal cross-cluster migration; and the *k* = 2 consensus matrix presented two high-consistency diagonal blocks. Accordingly, *k* = 2 was selected.

Cluster profiles (**Figure 10E**): Cluster 2—enriched for phlegm-dampness/damp-heat with qi stagnation and spleen deficiency, characterized by greasy tongue coating, sticky/bitter taste, abdominal fullness/pain, belching, wiry/slippery pulse, red/enlarged tongue with tooth marks, poor appetite, fatigue, loose stools, obesity; cluster 1—lighter overall symptom burden (relatively mild phenotype). The two clusters complement each other along a phlegm-dampness → damp-heat → qi stagnation → spleen deficiency axis, with strong clinical interpretability.

### Phenotype-stratified efficacy and formula prioritization

**Figure 11:**
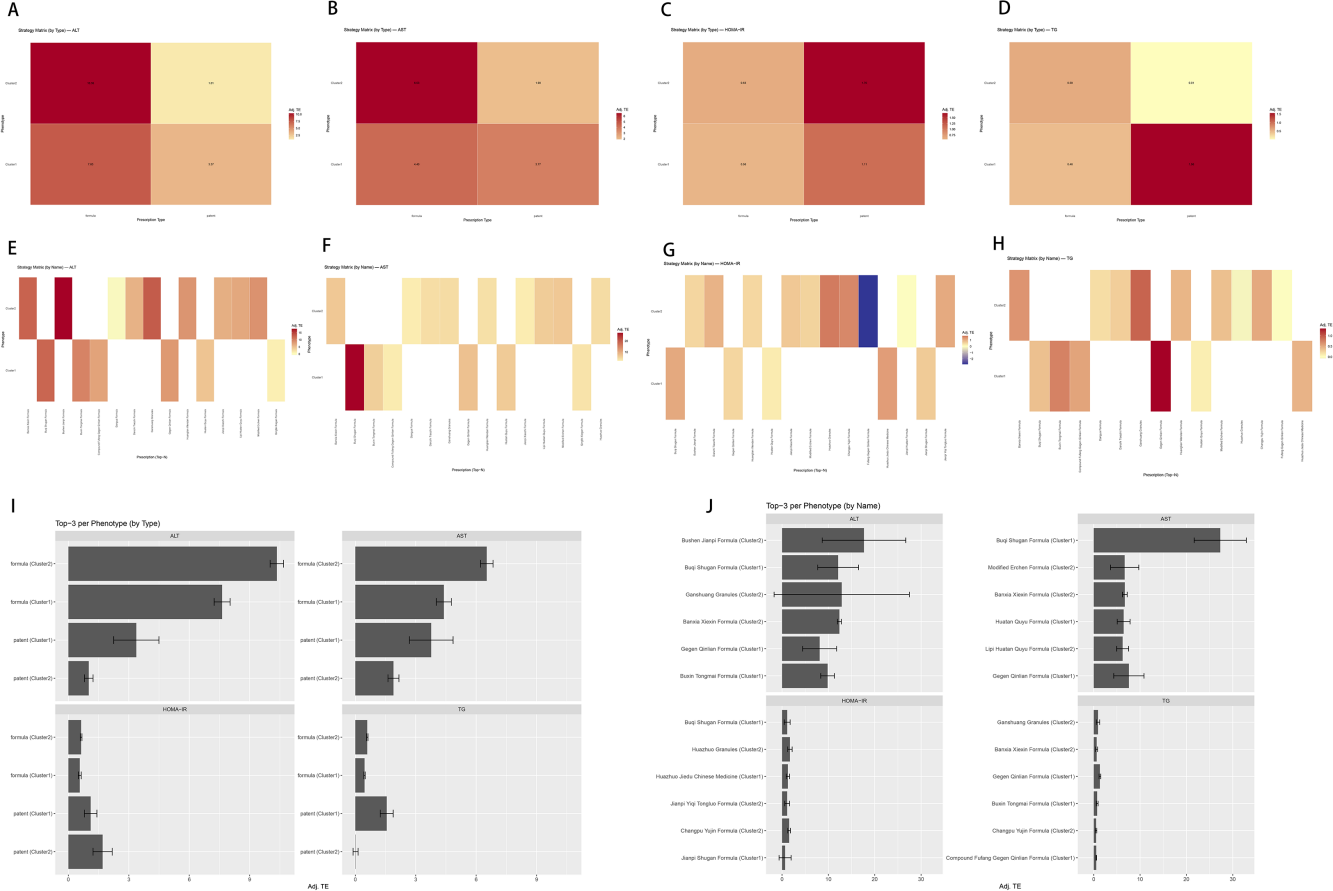
Phenotype-stratified intervention matrix and representative formulas. **(A–D)** Type×phenotype×outcome matrices (ALT, AST, HOMA-IR, TG): color = intensity of adjusted effect (Adj. TE). ALT/AST: classical formulas outperform in both phenotypes; HOMA-IR: Chinese patent medicines are superior in cluster 2; TG: prioritization varies by phenotype. **(E–H)** Representative formula-level matrices (selected). **(I)** Top 3 per phenotype by intervention type (classical formula/Chinese patent medicine). **(J)** Top 3 per phenotype by formula name (with error bars). Cluster 1 = lower symptom burden; cluster 2 = higher burden with phlegm-dampness/damp-heat.

ALT/AST (**Figures 11A, B**): In both clusters, classical formulas outperformed CPMs, with a larger advantage in cluster 1. HOMA-IR (**Figure 11C**): The overall effect was small; CPMs were relatively more effective in cluster 2; no significant difference in cluster 1. TG (**Figure 11D**): Clear phenotype dependence: in cluster 1, CPMs were superior; in cluster 2, classical formulas showed moderate efficacy, whereas CPMs were weaker. Top formulas (**Figures 11E–J**): The top three representatives per phenotype aligned with the mini-meta analyses, providing evidence-based prioritization of formula types by phenotype.

### Predictive performance and interpretable drivers

Model performance (**Figures 2A–C**): Stratified machine learning on the trial–phenotype–intervention dataset showed modest discrimination with OOF ROC-AUC = 0.590 (**Figure 2A**) and favorable precision–recall characteristics (OOF PR-AUC = 0.903; **Supplementary Table 11**), with acceptable calibration (Brier = 0.112; 10-bin ECE = 0.065; **Supplementary Material S2**, **Supplementary Table 11**; calibration curve in **Figure 2C**). Sensitivity analyses designed to reduce study-level leakage and assess robustness were directionally consistent (LOSO-like holdout and subset checks; **Supplementary Material S2**, **Supplementary Tables 12**, **S13**).

Global importance and marginal effects (**Figures 2D–K**). Larger n_treat/n_control, longer follow-up, smaller SE_TE, and add-on design were consistently associated with a higher probability of improvement, displaying a small-sample amplification → mid-sample plateau pattern that aligns with weighted regression and inflection-point signals (≥40–50 per group). SE_TE showed a monotonic negative association with improvement probability. Individual SHAP plots reproduced these trends (**Supplementary Figure 11**).

Driver consistency and response spectrum (**Supplementary Figure 12**). Across models and outcomes, the top 10/20 drivers were highly consistent. The “response spectrum” heatmap suggested a learnable coupling among phenotype, formula, and outcome—for example, for TG, *Salvia miltiorrhiza*, *Alisma orientale*, and features related to phlegm-dampness/damp-heat/liver depression with spleen deficiency correlated positively with improvement, with a few negatively correlated items also observed (**Supplementary Figures 12J, K**).

## Discussion

We distill heterogeneous RCT evidence into three evidence-derived, hypothesis-generating rules (summarized in **Figure 3**): (A) Under TE harmonization, the pooled effects for ALT/AST, HOMA-IR, and TG are directionally favorable, but between-study variability is substantial and 95% prediction intervals for several biochemical endpoints are wide and frequently cross the null. In the add-on primary set, CAP provides a comparatively more reproducible short-term imaging signal with low heterogeneity and a PI that does not cross the null (**Supplementary Material 2**, **Supplementary Table 7**); (B) Design features such as ≥40–50 participants per arm and ≥12–16-week follow-up were associated with improved reproducibility/precision of estimates in the published evidence landscape; however, these patterns may reflect correlated design and reporting features (e.g., small-study effects and selective reporting) and should be treated as evidence-derived, hypothesis-generating signals rather than causal thresholds; (C) Time-window alignment matters: prioritize CAP for early windows and reserve LSM for longer follow-up. These rules together translate synthesis into trial-planning anchors and cautious, clinically interpretable guidance that warrants prospective validation. These “rules” summarize empirical patterns in the available evidence and are intended for trial planning and cautious interpretation rather than prescriptive clinical decision-making.

Most prior studies emphasized herb–component–target or co-occurrence patterns that are difficult to align with clinical trial evidence and TCM phenotypes (17–19). Anchored in RCTs, our work restructures the evidence as a formula–syndrome–symptom–outcome–safety map, yielding a visual, comparable, and searchable KG. Importantly, the KG is used to organize and navigate evidence rather than to infer causality: apparent differences across interventions can largely reflect reporting architecture—numbers of trials, add-on design, background Western medications, follow-up window, and endpoint preferences—so we refrain from causal claims. Safety underreporting: Safety reporting is also part of this reporting architecture and was frequently incomplete: over half of the included trials did not report adverse events (**Supplementary Material 2**, **Supplementary Table 1**, column BH). Therefore, safety observations in this review apply only to the subset of trials with explicit AE information and should not be extrapolated as “overall safety.” Future RCTs should follow CONSORT harms reporting and explicitly report AE monitoring methods and results even when no events occur. To further mitigate this concern, we explicitly assessed background-therapy comparability for mixed trials and coded it as balanced/unclear/imbalanced (**Supplementary Material 2**, **Supplementary Table 1**, column AZ), and we interpret mixed findings accordingly as exploratory. Along the syndrome → symptom → formula pathway, common syndromes (e.g., liver depression and spleen deficiency; intermingled phlegm–stasis) link to practical diagnostic clues (lassitude, poor appetite, abdominal distension, tooth-marked tongue) and then to formula categories, providing trial-traceable anchors for syndrome discrimination in practice and for protocol planning (19).

The herb–herb network reveals a three-cluster structure built on a universal base (*Pinellia*–*Poria*–*Citrus reticulata*–*Atractylodes macrocephala*–*Glycyrrhiza*), with functional modules (e.g., *Salvia*/*Bupleurum*/*Coptis*; *Scutellaria*/*Astragalus*; *Codonopsis*/*Crataegus*) added as needed. Dosage patterns suggest that most base herbs are used at moderate doses. While these modules do not by themselves imply efficacy differences, they provide objective, pre-matchable building blocks that can support transparent reporting and enable consistent intervention specification when aligning formula design with target outcomes.

Consistent with earlier systematic reviews and meta-analyses reporting improvements in liver enzymes, blood lipids, and insulin resistance under TCM for MASLD—while also noting unstable effect sizes and substantial heterogeneity (10, 17, 20, 21) —our unified metric (TE > 0 = improvement) recovers coherent benefits in ALT, AST, HOMA-IR, and TG. Notably, the prediction intervals for these biochemical endpoints are wide and often cross the null (**Supplementary Tables 7**, **S8**), indicating that the magnitude—and in some settings even the presence—of benefit may vary across future individual trials despite directionally favorable pooled means. To address concerns about clinical heterogeneity across “TCM,” we additionally synthesized primary endpoints within prespecified intervention strata (classic formulas, custom formulas, patent medicines, and single-herb/extracts; **Supplementary Material 2**, **Supplementary Table 6**). Pooled effects were directionally favorable across major strata, but substantial residual heterogeneity persisted, and evidence for single-herb/extract interventions was sparse and therefore treated as exploratory. Several systematic reviews and meta-analyses have been published in this area. Epidemiologic SR/MAs have quantified the high burden of MASLD in patients with T2DM at the population level (7, 19, 22). In parallel, SR/MAs evaluating TCM/Chinese herbal medicine in T2DM with MASLD have generally reported improvements in liver enzymes, lipid profiles, and insulin-resistance-related indices while also emphasizing recurring evidence limitations—heterogeneous trial designs, non-uniform background therapy, variable endpoint definitions, and imprecision driven by small samples and short follow-up (10, 23). These interpretations are consistent with the GRADE Summary of Findings, in which certainty for primary outcomes was commonly downgraded due to inconsistency and/or imprecision (**Supplementary Material 2**, **Supplementary Table 9**).

Compared with these prior SR/MAs, our study differs in both scope and analytical deliverables. First, we reorganize RCT evidence into an RCT-anchored, multi-layer knowledge graph linking formula–syndrome/symptom–outcome–safety, enabling navigation at the “phenotype × target outcome” level rather than reporting pooled averages alone. Second, we implement unified effects (TE > 0 = improvement) to harmonize effect direction across heterogeneous endpoints and pre-specify a cross-domain endpoint set (liver injury, metabolism, lipids, and imaging), thereby reducing pseudo-heterogeneity and yielding a coherent outcome fingerprint. Third, beyond conventional pooling, we evaluate how key design features (including sample size per arm and follow-up duration) relate to effect-estimate stability using weighted meta-regression and interpretable machine learning (SHAP/ALE), providing reproducibility-oriented guidance for future trial planning. To minimize study-level leakage and to frame these ML-derived signals as exploratory, models were evaluated using study-grouped fivefold OOF predictions and additional robustness checks (**Supplementary Material 2**, **Supplementary Table 11**-**S13**). Finally, we stratified synthesis by trial design (add-on vs. mixed) to improve interpretability under non-uniform background therapy—a limitation repeatedly noted in earlier syntheses (10, 19, 23). Primary efficacy inferences are drawn from the add-on stratum, where background WM regimens are balanced by design, whereas mixed trials are treated as exploratory/sensitivity evidence given variable or incompletely reported co-interventions (**Supplementary Table 1**, column AZ).

A quantitative observation from the available evidence is that pooled signals tend to be more reproducible and/or more precise when *n* ≥ 40–50 per arm and follow-up ≥12–16 weeks, although this pattern is exploratory and may be influenced by correlated design and reporting features. Below these thresholds, statistical uncertainty (SE) itself reduces the probability of detecting improvement, implying that much prior “instability” reflects low signal-to-noise rather than absent effects (17, 18). Weighted meta-regression and SHAP/ALE converged on this empirical pattern, which should be interpreted as hypothesis-generating rather than causal.

Because add-on designs dominate and can yield “easier” improvements, extrapolation to single-agent use is often unclear (19, 24). While add-on improves SNR and treatment consistency, it may mask marginal effects of individual agents; pooling across differing background intensities can also confound estimates. We therefore recommend prespecified design stratification and sensitivity analyses at the protocol stage as design anchors. Accordingly, we base the principal clinical interpretation on add-on trials and use mixed trials primarily to assess directional consistency under less controlled background therapy.

Regarding imaging endpoints, many studies used LSM as a primary short-term endpoint and reported unstable or null findings over 12–24 weeks (25–27). Our chain of evidence indicates that CAP is more sensitive to near-term hepatic fat change, whereas LSM is influenced by inflammation/congestion and better reflects fibrosis on a delayed time window. Prioritizing CAP and metabolic indices short-term, while reserving LSM for longer-term evaluation, may reduce false negatives caused by time-window mismatch. This interpretation is consistent with our interval evidence: CAP shows a PI that remains below the null, whereas LSM exhibits a PI crossing the null over short follow-up (**Supplementary Table 7**), supporting a cautious interpretation of short-term stiffness changes.

Unlike most reviews that emphasize overall pooled average efficacy (17, 28), we translate synthesis into actionable stratification via clustering and a strategy matrix: when the targets are ALT/AST and TG, classical formulas aimed at clearing turbidity/resolving dampness and regulating qi/harmonizing the middle tended to show directionally favorable pooled signals for enzyme/lipid targets in the available add-on evidence; when the target is HOMA-IR, custom formulas provide broader coverage; in phenotypes with high damp-heat burden, Chinese patent medicines are an optional strategy. These patterns are prioritization cues rather than evidence of superiority, given residual heterogeneity and wide prediction intervals. These guide target × phenotype prioritization without asserting causality or mechanism. Notably, our main synthesis excludes single-herb/extract trials due to sparse evidence, and category-specific signals are supported by sensitivity analyses restricted to formulas only (classic + custom) and excluding single-herb/extracts, which yielded consistent overall patterns (**Supplementary Table 6**). Accordingly, these category-linked suggestions are intended for prioritization and trial planning, not for ranking individual interventions.

Methodologically, to address inconsistent directional semantics, mixed units, and coarse stratification (29, 30), we (i) unified directions/units with TE >0 = improvement to mitigate pseudo-heterogeneity, (ii) extended beyond random-effects pooling by using weighted meta-regression to characterize sample-size/follow-up thresholds, and (iii) employed SHAP/ALE to corroborate the nonlinear pattern (↑SE → ↓improvement probability) and to integrate phenotype × formula category × outcome stability into a navigable matrix. The practical question thus becomes “under which conditions and populations are improvements most likely?”—increasing executability. Where trial-design interpretations are involved, these prioritization cues are anchored primarily in add-on evidence and supported by mixed analyses only as exploratory context.

Compared with prior work (31, 32), the added value is not asserting one formula “best,” but offering an implementable framework. Clinically, first, fix the target outcome (e.g., ALT/AST, TG, HOMA-IR) and phenotype (phlegm-dampness/damp-heat vs. liver-depression/spleen-deficiency burden) and then select formula categories. In research, consider adequately powered designs, and if feasible, aim for *n* ≥ 40–50 per arm with ≥12–16 weeks of follow-up; use CAP/metabolic endpoints short-term and add LSM long-term; distinguish add-on intensity with prespecified stratification/sensitivity. These recommendations are evidence-anchored at the outcome level and require prospective validation rather than mechanistic extrapolation.

## Conclusion

Anchored in RCT evidence, we built a formula–syndrome/symptom–outcome–safety knowledge graph to support searchable, evidence-ready navigation and trial-planning prioritization. Primary inferences were based on add-on trials with balanced background Western medicine, while mixed designs were interpreted as exploratory. Under this framework, pooled effects for ALT/AST, HOMA-IR, and TG were directionally favorable after TE harmonization, but between-study variability was substantial and prediction intervals often spanned null, indicating that future individual trials could observe attenuated or null effects. CAP provided a comparatively more reproducible short-term imaging signal, whereas short-term LSM remained variable, suggesting that imaging interpretation is sensitive to follow-up window alignment. Evidence-derived design patterns (e.g., larger sample sizes and longer follow-up) and phenotype-guided signals should be regarded as hypothesis-generating rather than causal thresholds, and safety inferences are constrained by substantial harms underreporting, with reported events mainly mild gastrointestinal or systemic symptoms.

## Data Availability

The original contributions presented in the study are included in the article/**Supplementary Material**. Further inquiries can be directed to the corresponding author.
