# Supplementary material for: Traditional Chinese medicine for type 2 diabetes with metabolic-associated steatotic liver disease: unified effects and design thresholds
Source: Front Endocrinol (Lausanne). 2026 Mar 3;17:1740126. doi: 10.3389/fendo.2026.1740126 (PMC12992033; doi:10.3389/fendo.2026.1740126)
Supplement: Supplementary file 14 [file DataSheet1.pdf]

Literature search formula

Time span 2015-01-01 to 2025-08-31

PubMed literature search formula：("type 2 diabetes"[Title/Abstract] OR "T2DM"[Title/Abstract]) AND("NAFLD"[Title/Abstract] OR "nonalcoholic fatty liver disease"[Title/Abstract]) AND("randomized controlled trial"[Publication Type] OR "randomized"[Title/Abstract])

Embase literature search formula：("Fatty Liver"[MeSH] OR "Nonalcoholic Fatty Liver Disease"[MeSH]OR NAFLD[All] OR MAFLD[All] OR MASLD[All]OR NASH[All]ORMASH[All]ORsteatohepatitis[All]ORhepaticsteatosis[All])AND("Medicine,ChineseTraditional"[MeSH]OR"Drugs,ChineseHerbal"[MeSH]OR"Phytotherapy"[MeSH]OR"Plant Extracts"[MeSH]OR "traditional Chinese medicine"[All] OR"Chinese herbal"[All] OR (herbal[All] AND (formula [All] OR decoction[All] OR granule [All] OR capsule [All]))OR "Chinese patent medicine"[All])

Web of Scienceliterature search formula：TS=(("nonalcoholic fatty liver disease" OR NAFLD OR "metabolic dysfunction-associated fatty liver disease" OR MAFLD OR "metabolic dysfunction-associated steatotic liver disease" OR MASLD OR "non-alcoholic steatohepatitis" OR NASH OR MASH OR steatohepatitis OR "hepatic steatosis") AND ("type 2 diabetes" OR T2DM OR "insulin resistance" OR HOMA-IR) AND ("traditional Chinese medicine" OR "Chinese herbal" OR "herbal formula" OR decoction OR "Chinese patent medicine" OR phytotherapy OR "plant extract"))

Chinese databases. We searched three Chinese bibliographic databases—CNKI, Wanfang, and VIP—from January 1, 2015 to August 31, 2025. Searches combined controlled fields equivalent to Topic/Subject (title, keywords, and abstract where applicable) for:

(1) fatty liver disease terms (NAFLD/MAFLD/MASLD; NASH/MASH; hepatic steatosis/fatty liver),

(2) type 2 diabetes terms (T2DM; insulin resistance; HOMA-IR), and

(3) traditional Chinese medicine (TCM).

No language filter was applied within Chinese databases. The exact Chinese queries executed are provided verbatim below for reproducibility.

CNKI (Topic field)

English description (logic): Topic = (NAFLD/MAFLD/MASLD/NASH/MASH/steatosis/fatty liver) AND Topic = (type 2 diabetes/T2DM) AND Topic = (TCM).

Original Chinese query used:主题: (非酒精性脂肪性肝病 OR NAFLD OR 代谢相关脂肪性肝病 OR MAFLD OR 代谢功能障碍相关脂肪性肝病 OR MASLD OR 非酒精性脂肪性肝炎 OR NASH OR MASH OR 肝脂肪变性 OR 脂肪性肝炎) AND 主题: (2 型糖尿病 OR Ⅱ型糖尿病 OR T2DM) AND 主题:(中医)

Wanfang (Topic field)

English description (logic): Topic = (NAFLD/MAFLD/MASLD/NASH/MASH/steatosis/fatty liver) AND Topic = (type 2 diabetes/T2DM) AND Topic = (TCM).

Original Chinese query used:主题: (非酒精性脂肪性肝病 OR NAFLD OR 代谢相关脂肪性肝病 OR MAFLD OR 代谢功能障碍相关脂肪性肝病 OR MASLD OR 非酒精性脂肪性肝炎 OR NASH OR MASH OR 肝脂肪变性 OR 脂肪性肝炎) AND 主题: (2 型糖尿病 OR Ⅱ型糖尿病 OR T2DM) AND 主题: (中医)

VIP (Topic field)

English description (logic): Topic = (NAFLD/MAFLD/MASLD/NASH/MASH/steatosis/fatty liver) AND Topic = (type 2 diabetes/T2DM/insulin resistance/HOMA-IR) AND Topic = (TCM).

Original Chinese query used:主题: (非酒精性脂肪性肝病 OR NAFLD OR 代谢相关脂肪性肝病 OR MAFLD OR 代谢功能障碍相关脂肪性肝病 OR MASLD OR 非酒精性脂肪性肝炎 OR NASH OR MASH OR 肝脂肪变性 OR 脂肪性肝炎)AND 主题: (2 型糖尿病 OR Ⅱ型糖尿病 OR T2DM OR 胰岛素抵抗 OR HOMA-IR) AND 主题: (中医)

In Chinese databases, searches were executed using Chinese keywords in the Topic/Subject field; for transparency, we report both the English description of the search logic and the verbatim Chinese queries.

The PRISMA 2020 checklist is provided in Supplementary Material 1 (Appendix)

PRISMA 2020 Checklist

Section and Topic	Item #	Checklist item	Location where item is reported
**TITLE**			
Title	1	Identify the report as a systematic review.	Title Page (Page 1)
**ABSTRACT**			
Abstract	2	See the PRISMA 2020 for Abstracts checklist.	Abstract (Page 1-3)
**INTRODUCTION**			
Rationale	3	Describe the rationale for the review in the context of existing knowledge.	Introduction, Paragraph 1–2 (Page 3-4)
Objectives	4	Provide an explicit statement of the objective(s) or question(s) the review addresses.	Introduction, Paragraph 3–4 (Page 4)
**METHODS**			
Eligibility criteria	5	Specify the inclusion and exclusion criteria for the review and how studies were grouped for the syntheses.	Methods - Eligibility Criteria (Page 5-6)
Information sources	6	Specify all databases, registers, websites, organisations, reference lists and other sources searched or consulted to identify studies. Specify the date when each source was last searched or consulted.	Methods - Literature Search (Page 5)
Search strategy	7	Present the full search strategies for all databases, registers and websites, including any filters and limits used.	Supplementary Material 1 (Full search strategy)
Selection process	8	Specify the methods used to decide whether a study met the inclusion criteria of the review, including how many reviewers screened each record and each report retrieved, whether they worked independently, and if applicable, details of automation tools used in the process.	Methods - Eligibility Criteria (Page 5)
Data collection process	9	Specify the methods used to collect data from reports, including how many reviewers collected data from each report, whether they worked independently, any processes for obtaining or confirming data from study investigators, and if applicable, details of automation tools used in the process.	Methods - Data Extraction (Page 6)
Data items	10a	List and define all outcomes for which data were sought. Specify whether all results that were compatible with each outcome domain in each study were sought (e.g. for all measures, time points, analyses), and if not, the methods used to decide which results to collect.	Methods – Meta-analysis（pre-specified primary endpoints + time window rules）/ Effect Size & Unified Effects (Page 6-8)
	10b	List and define all other variables for which data were sought (e.g. participant and intervention characteristics, funding sources). Describe any assumptions made about any missing or unclear information.	Methods – Data Extraction and Standardization(Page 6-8) + trial-level variables in Supplementary Material 2 Table S1
Study risk of bias assessment	11	Specify the methods used to assess risk of bias in the included studies, including details of the tool(s) used, how many reviewers assessed each study and whether they worked independently, and if applicable, details of automation tools used in the process.	Methods – Risk of bias and certainty of evidence（RoB 2 + decision rules）(Page 10)+ Supplementary Table S10（RoB-2 rules）
Effect measures	12	Specify for each outcome the effect measure(s) (e.g. risk ratio, mean difference) used in the synthesis or presentation of results.	Methods – Meta-analysis（MD / REML random-effects）+ Effect Size / Unified Effects(Page9-11)

PRISMA

PRISMA 2020 Checklist

Section and Topic	Item #	Checklist item	Location where item is reported
Synthesis methods	13a	Describe the processes used to decide which studies were eligible for each synthesis (e.g. tabulating the study intervention characteristics and comparing against the planned groups for each synthesis (item #5)).	Methods - Statistical Analysis & Meta-regression (Page 9-11)
	13b	Describe any methods required to prepare the data for presentation or synthesis, such as handling of missing summary statistics, or data conversions.	Methods – Data Extraction and Standardization (Page 6-8)
	13c	Describe any methods used to tabulate or visually display results of individual studies and syntheses.	Methods – Knowledge Graph Construction + Visualization/figures description (Page 8-9) + Supplementary tables (S1–S5)
	13d	Describe any methods used to synthesize results and provide a rationale for the choice(s). If meta-analysis was performed, describe the model(s), method(s) to identify the presence and extent of statistical heterogeneity, and software package(s) used.	Methods – Meta-analysis（REML random-effects ，I^2/τ^2/PI，R） (Page 9-11)
	13e	Describe any methods used to explore possible causes of heterogeneity among study results (e.g. subgroup analysis, meta-regression).	Methods – Meta-analysis（design stratification / subgroup） + Weighted Meta-regression(Page 9-11)
	13f	Describe any sensitivity analyses conducted to assess robustness of the synthesized results.	Methods – Meta-analysis（leave-one-out/top-Δ/Baujat）(Page 9-11)+ Supplementary Tables S7–S8 / figures S4–S8
Reporting bias assessment	14	Describe any methods used to assess risk of bias due to missing results in a synthesis (arising from reporting biases).	Methods – Meta-analysis(Page 9-11)
Certainty assessment	15	Describe any methods used to assess certainty (or confidence) in the body of evidence for an outcome.	Methods – Risk of bias and certainty of evidence（GRADE Page 10） + Supplementary Table S9（SoF/GRADE）
**RESULTS**			
Study selection	16a	Describe the results of the search and selection process, from the number of records identified in the search to the number of studies included in the review, ideally using a flow diagram.	Results（Basic Characteristics)(Page 12-14)+ Fig.1 PRISMA flow diagram
	16b	Cite studies that might appear to meet the inclusion criteria, but which were excluded, and explain why they were excluded.	Supplementary Material S2-TableS1
Study characteristics	17	Cite each included study and present its characteristics.	Results – Basic Characteristics)(Page 12-14) + Table 1 + Supplementary Material 2 Table S1（trial-level summary）
Risk of bias in studies	18	Present assessments of risk of bias for each included study.	Results – Meta-analysis(Page 17-20) + Fig. S1 + Supplementary Material 2 Table S1 (BI–BO)

PRISMA

PRISMA 2020 Checklist

Section and Topic	Item #	Checklist item	Location where item is reported
Results of individual studies	19	For all outcomes, present, for each study: (a) summary statistics for each group (where appropriate) and (b) an effect estimate and its precision (e.g. confidence/credible interval), ideally using structured tables or plots.	Forest plots:Fig. 7 / Fig. S4
Results of syntheses	20a	For each synthesis, briefly summarise the characteristics and risk of bias among contributing studies.	Results – Meta-analysis(Page 17-20)
	20b	Present results of all statistical syntheses conducted. If meta-analysis was done, present for each the summary estimate and its precision (e.g. confidence/credible interval) and measures of statistical heterogeneity. If comparing groups, describe the direction of the effect.	Results – Add-on therapys(Page 18-19)（Fig.7，MD/CI/I²/PI ）+ Mixed（Fig.S4）
	20c	Present results of all investigations of possible causes of heterogeneity among study results.	Results – Meta-regression(Page 20-21) + subgroup（Fig.8 / Fig.S9）
	20d	Present results of all sensitivity analyses conducted to assess the robustness of the synthesized results.	Results – Bias and robustness(Page 19-20)（Baujat/leave-one-out/top-Δ）+ Figs S5–S8
Reporting biases	21	Present assessments of risk of bias due to missing results (arising from reporting biases) for each synthesis assessed.	Results – Bias and robustness(Page 19-20)（funnel plots；Fig. S4G–K）
Certainty of evidence	22	Present assessments of certainty (or confidence) in the body of evidence for each outcome assessed.	Results – Risk of bias and certainty of evidence（GRADE)(Page 18 and Page 19-20)+ Supplementary Table S9（SoF/GRADE）
**DISCUSSION**			
Discussion	23a	Provide a general interpretation of the results in the context of other evidence.	Discussion - Paragraph 1
	23b	Discuss any limitations of the evidence included in the review.	Discussion - Limitations
	23c	Discuss any limitations of the review processes used.	Discussion – Limitations of review processes
	23d	Discuss implications of the results for practice, policy, and future research.	Discussion – Implications
**OTHER INFORMATION**			
Registration and protocol	24a	Provide registration information for the review, including register name and registration number, or state that the review was not registered.	Methods—Reporting and Registration（PROSPERO：CRD420251167450）
	24b	Indicate where the review protocol can be accessed, or state that a protocol was not prepared.	Protocol not publicly posted; available upon request.
	24c	Describe and explain any amendments to information provided at registration or in the protocol.	No amendments to the registered protocol were made.
Support	25	Describe sources of financial or non-financial support for the review, and the role of the funders or sponsors in the review.	Declarations— No Funding
Competing interests	26	Declare any competing interests of review authors.	Declarations—Conflicts of interest（No competing

PRISMA 2020 Checklist

Section and Topic	Item #	Checklist item	Location where item is reported
			interests）
Availability of data, code and other materials	27	Report which of the following are publicly available and where they can be found: template data collection forms; data extracted from included studies; data used for all analyses; analytic code; any other materials used in the review.	Data Availability Statement (Supplementary Files)

From: Page MJ, McKenzie JE, Bossuyt PM, Boutron I, Hoffmann TC, Mulrow CD, et al. The PRISMA 2020 statement: an updated guideline for reporting systematic reviews. BMJ 2021;372:n71. doi: 10.1136/bmj.n71. This work is licensed under CC BY 4.0. To view a copy of this license, visit https://creativecommons.org/licenses/by/4.0/
